# Roles of the intestinal microbiota and microbial metabolites in acute GVHD

**DOI:** 10.1186/s40164-021-00240-3

**Published:** 2021-10-27

**Authors:** Dandan Lin, Bo Hu, Pengfei Li, Ye Zhao, Yang Xu, Depei Wu

**Affiliations:** 1 National Clinical Research Center for Hematologic Diseases, Jiangsu Institute of Hematology, The First Affiliated Hospital of Soochow University, 188 Shizi Street, Suzhou, 215006 People’s Republic of China; 2 Institute of Blood and Marrow Transplantation, Collaborative Innovation Center of Hematology, Soochow University, Suzhou, 215123 People’s Republic of China

**Keywords:** Acute GVHD, Intestinal microbiota, Metabolites, Microbiota intervention

## Abstract

Allogeneic hematopoietic stem cell transplantation (allo-HSCT) is one of the most curative strategies for the treatment of many hematologic malignancies and diseases. However, acute graft-versus-host disease (GVHD) limits the success of allo-HSCT. The prevention and treatment of acute GVHD is the key issue for improving the efficacy of allo-HSCT and has become a research hotspot. The intestine is the primary organ targeted by acute GVHD, and the intestinal microbiota is critical for maintaining the homeostasis of the intestinal microenvironment and the immune response. Many studies have demonstrated the close association between the intestinal microbiota and the pathogenesis of acute GVHD. Furthermore, dysbiosis of the microbiota, which manifests as alterations in the diversity and composition of the intestinal microbiota, and alterations of microbial metabolites are pronounced in acute GVHD and associated with poor patient prognosis. The microbiota interacts with the host directly via microbial surface antigens or microbiota-derived metabolites to regulate intestinal homeostasis and the immune response. Therefore, intervention strategies targeting the intestinal microbiota, including antibiotics, prebiotics, probiotics, postbiotics and fecal microbiota transplantation (FMT), are potential new treatment options for acute GVHD. In this review, we discuss the alterations and roles of the intestinal microbiota and its metabolites in acute GVHD, as well as interventions targeting microbiota for the prevention and treatment of acute GVHD.

## Background

Allogeneic hematopoietic stem cell transplantation (allo-HSCT) is one of the most curative strategies for the treatment of many hematologic malignancies and immunological diseases [1, 2]. After transplantation, allogeneic donor T cells can attack residual malignant cells to exert graft-versus-leukemia (GVL) effects, and they can also attack the target organs and tissues of the host to cause graft-versus-host disease (GVHD). Approximately 30–50% of allo-HSCT patients develop acute GVHD (aGVHD), and approximately 14% of patients develop severe aGVHD (grade III-IV), limiting the success of allo-HSCT and seriously worsening the survival and prognosis of patients. The one-year survival rate of patients with severe aGVHD is only 40% [3, 4].

**Fig. 1:**
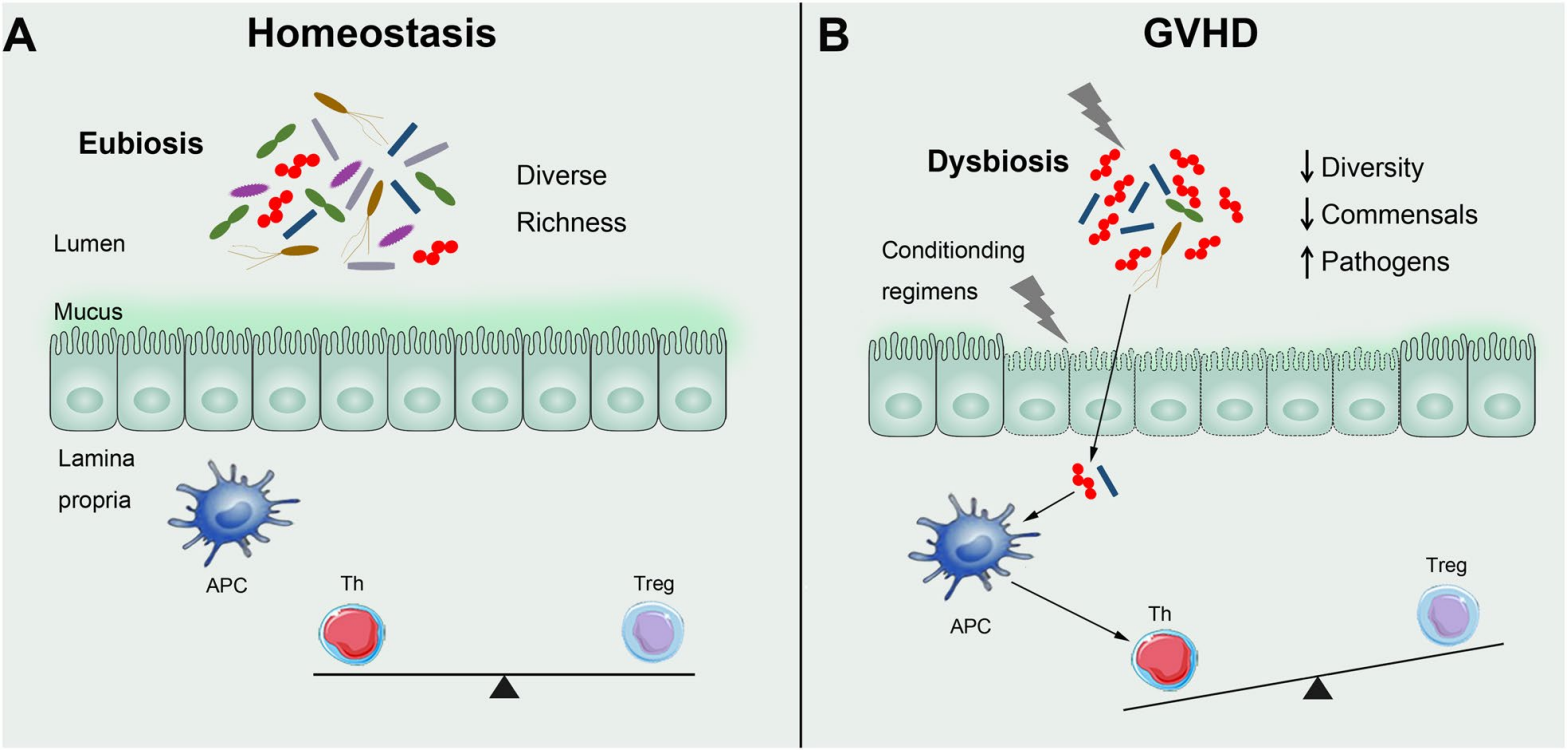
Overview of intestinal microbiota and intestinal homeostasis. **A** In the healthy state, the intestinal barrier maintains its integrity and prevents translocation of luminal microbiota. The interaction between microbiota, immunity and intestinal barrier is in a steady state maintaining intestinal homeostasis. **B** During allo-HSCT, conditioning regimens (including irradiation, chemotherapy and antibiotics) damage the gastrointestinal tract and result in dysbiosis of the intestinal microbiota. The destroy of the intestinal barrier integrity permits translocation of bacteria across the barrier. The recognition of DAMPs/MAMPs by antigen presenting cells (APCs) induces pro-inflammatory response including activation of T cells and cytokine storm to aggravate tissue damage and promote GVHD development. Th, T help cells; Treg, regulatory T cells; APC, antigen presenting cell

The gastrointestinal (GI) tract is a primary target organ of aGVHD, and GI GVHD can cause a series of complications (infection, bleeding, etc.) that aggravate systemic GVHD and increase mortality [5]. The human gastrointestinal tract is colonized by abundant, highly diverse and complex commensal microbial species, including bacteria, archaea, fungi, protozoa, and viruses. More than 1000 microbial species reside in the intestines of healthy individuals, and the microbial density in fecal matter is approximately 10^13^–10^14^ cells/g [6, 7]. The commensal intestinal microbiota is substantially beneficial to humans, as it regulates metabolic processes, maintains intestinal homeostasis and epithelial integrity, protects against pathogens, and modulates immune system development and the immune response. The intestinal barrier, a dense structure composed of a monolayer of intestinal epithelial cells (IECs), interacts with intestinal microbes [8, 9]. Host immune cells reside in the intestinal lamina propria, which is located below the epithelial layer. The numerous microbes in the intestinal cavity provide a special microenvironment for immune cells in the GI tract. The crosstalk between the intestinal microbiota and immune cells in this microenvironment is key to the maintenance of intestinal homeostasis (Fig. 1) [10, 11]. Intestinal barrier damage is the initial step in the development of aGVHD, as it permits the translocation of bacteria across the barrier, which disrupts intestinal immune homeostasis [12].

In this review, we discuss recent progress related to the roles of the intestinal microbiota and its metabolites in aGVHD, as well as interventions targeting the microbiota for the prevention and treatment of aGVHD.

### Intestinal microbiota dysbiosis and aGVHD

In the 1970s, the absence of the intestinal microbiota was reported to mitigate aGVHD in germ-free mice, indicating the importance of the intestinal microbiota in the occurrence and development of aGVHD [13, 14]. In 1992, a study on human patients showed that the suppression of the intestinal anaerobic bacterial microbiota reduced the risk of aGVHD [15]. Along with the interaction between intestinal homeostasis and microbiota dynamics, the role of the intestinal microbiota in aGVHD is receiving more attention.

Healthy individuals have diverse and stable intestinal microbiotas that are mainly composed of the phyla *Firmicutes* and *Bacteroidetes*, which account for ∼90% of the microbiota. The low-abundance phyla in the intestinal microbiota include *Actinobacteria*, *Proteobacteria*, *Verrucomicrobia* and *Fusobacteria* [16]. In allo-HSCT patients, conventional therapeutic regimens (chemotherapy, radiotherapy or antibiotics) damage the gastrointestinal tract and cause intestinal microbiota dysbiosis [17, 18]. Loss of intestinal microbial diversity and alteration of the microbial composition are found in allo-HSCT patients and are more pronounced in aGVHD patients. Therefore, the microbiota may serve as a predictor of aGVHD in allo-HSCT patients [19–21].

### Loss of microbial diversity in aGVHD

Microbial diversity was shown to be dramatically reduced in aGVHD patients and mouse models [18, 22]. Taur et al. examined the microbial diversity of allo-HSCT recipients using bacterial 16S rRNA gene sequence analysis, and the patients were divided into low-, intermediate-, and high-diversity groups based on the inverse Simpson index. The overall survival (OS) at three years was 36% for the low-diversity group, 60% for the intermediate-diversity group, and 67% for the high-diversity group (p = 0.019). Furthermore, the transplant-related mortality (TRM) rates were 53%, 23%, and 9% for the low-, intermediate- and high-diversity groups, respectively (p = 0.003). In addition, death due to GVHD or infection was also more frequent in the low-diversity group [23]. By analyzing the association between intestinal microbial diversity and mortality using fecal samples from 1362 allo-HSCT patients at four centers, a recently published study showed that higher diversity was associated with lower mortality, while lower diversity was associated with higher GVHD related mortality [20]. Moreover, high donor microbial diversity is linked to a lower risk of GI aGVHD [24]. Other studies have also reported correlations between the intestinal microbial diversity and the incidence and mortality of aGVHD (summarized in Table 1), and these findings were recently confirmed by a meta-analysis [25]. Together, these results indicate that the diversity of the intestinal microbiota is an important factor regulating allo-HSCT, as reduced diversity is associated with a poor overall survival, an increased risk of infection, a high risk of aGVHD and increased aGVHD-related mortality rates.

**Table 1:** Loss of intestinal microbial diversity is associated with aGVHD

Patients	Results	Microbiota analysis methods	References
Human/Mouse 18 adult patients	Loss of flora diversity was associated with GVHD in humans and mice	16S rRNA sequencing	[18]
Mouse	Diversity of the microbiota was significantly reduced in mice with GVHD	16S rRNA sequencing	[22]
Human 80 adult patients	Low intestinal microbiota diversity was associated with high TRM, lower OS, and GVHD-related mortality	16S rRNA sequencing	[23]
Human 64 adult patients	Increased bacterial diversity was linked to reduced aGVHD-related mortality	16S rRNA sequencing	[26]
Human 57 adult recipients and 22 paired adult donors	Recipients with lower diversity had a higher mortality; higher bacterial donor diversity was associated with lower risk of the acute GI GvHD	16S rRNA sequencing	[24]
Human 96 adult patients	Low bacterial α-diversity at 10 days after transplantation was significantly correlated with an increased risk of aGvHD	16S rRNA sequencing	[27]
Human 66 adult patients	Lower α-diversity of the stool microbiota was associated with aGVHD	16S rRNA sequencing	[28]
Human 81 adult patients	aGVHD patients had lower microbial diversity than non-aGVHD patients	16S rRNA sequencing	[29]
Human 141 adult patients	Microbial diversity was lower in aGVHD group than non-aGVHD group	16S rRNA sequencing	[21]
Human 10 pediatric patients	Gut microbial diversity showed a downward trend in children with GVHD	16S rRNA sequencing	[30]
Human 44 adult patients	Microbial diversity was associated with increased incidence of acute GI GVHD. Fecal butyrate and indole levels were correlated with microbial diversity	16S rRNA sequencing	[31]
Human 1362 adult patients	Lower diversity was associated with higher GVHD-related mortality	16S rRNA sequencing	[20]
Human 70 adult patients	Bacterial biomass and α-diversity were lower in severe aGVHD	16S rRNA sequencing	[32]
Human 150 adult patients	Low diversity was associated with high risk of aGVHD	shotgun metagenomic sequencing	[33]
Human 100 adult patients	Low α-diversity was significantly associated to increased risk of grade II-IV and III-IV acute GvHD	16S rRNA sequencing	[19]
Human 56 pediatric patients	Gut microbial diversity was lowest in GI aGVHD patients, which was consistent with higher mortality	16S rRNA sequencing	[34]

### Alteration of the microbial composition in aGVHD

Microbiota dysbiosis during allo-HSCT also manifests as a shift in the microbial composition. *Lactobacillales* (including *Enterococcus*, *Streptococcus* and *Lactobacillus* spp.), *Staphylococcaceae*, *Enterobacteriales* (including *Escherichia*, *Kluyvera*, *Klebsiella*, and *Enterobacter* spp.) and *Pasteurellales* are commonly expanded in aGVHD.

*Lactobacillales*, which belongs to the phylum *Firmicutes*, was shown to be expanded in subjects with aGVHD. Depletion of *Lactobacillales* with ampicillin in mice aggravated GVHD, while reintroduction of *Lactobacillus johnsonii* alleviated GVHD [18]. Conversely, another study reported that patients with a high abundance of *Lactobacillaceae* developed severe aGVHD and had increased mortality rates [35]. The expansion of *Enterococcus*, also belonging to the order *Lactobacillales*, was reported to be associated with aGVHD and to increase the severity and mortality of aGVHD in humans and mice [33, 35–40]. Vancomycin-resistant *Enterococcus* (VRE) and aerobic gram-negative bacteria are considered as the most common bacterial pathogens causing bloodstream infection [37, 41]. Moreover, *Enterococcus* growth is known to be dependent on lactose, and depletion of lactose reduces the abundance of *Enterococcus* and mitigates GVHD in mice. Allo-HSCT patients harboring the lactose malabsorption allele showed prolonged *Enterococcus* domination after antibiotic treatment, while the cumulative incidence of aGVHD in patients of this genotype was not significantly different [39]. The opposing role of *Lactobacillales* in aGVHD may be due to the different species that belong to this order. *Lactobacillus* exerts a protective effect, while *Enterococcus* aggravates the development of aGVHD. Additionally, the abundance of *Staphylococcaceae* within the phylum *Firmicutes* was increased during early onset in aGVHD patients [27].

Higher abundances of *Enterobacteriales* and *Pasteurellales* belonging to the phylum *Proteobacteria* have also been observed in aGVHD. Abundant *Enterobacteriaceae* is positively correlated with GVHD-related mortality [23]. The abundance of *Escherichia coli*, which belongs to the order *Enterobacteriales*, is expanded in mice with aGVHD and related to GVHD severity [22, 37]. Oral administration of polymyxin B can inhibit the expansion of *Escherichia coli* and improve GVHD in mice [22]. *Pasteurellales* is substantially abundant in pediatric patients with GI aGVHD and correlated with diarrhea severity [34].

Conversely, the abundances of *Clostridiales* (*Clostridium* spp., *Faecalibacterium* spp., *Lachnospiraceae* including *Blautia* and *Lachnoclostridium* spp., *Ruminococcaceae* including *Ruminococcus* spp., *Eubacteriaceae* including *Eubacterium* spp., and *Peptostreptococcaceae*), *Bacteroidetes* (including *Parabacteroides* and *Bacteroides*), and *Actinomycetaceae* were shown to be decreased in subjects with aGVHD.

In mouse models and patients with aGVHD, a reduced abundance of *Clostridiales* was observed in the intestinal microbiota, whereas the change in *Clostridiales* was not significant in non-GVHD patients [18, 36, 37]. Depletion of *Clostridia* is associated with the development of GVHD in humans and mice [42]. Administration of *Clostridia* spp. can mitigate GVHD and reduce mortality in mice [42, 43]. The higher abundance of *Lachnospiraceae* ameliorates aGVHD [32, 44]. Consistently, the decrease in *Blautia* spp., which belongs to the family *Lachnospiraceae*, also exacerbates aGVHD, while a higher abundance of *Blautia* spp. is associated with a reduced risk of aGVHD, decreased GVHD-related mortality and improved overall survival in humans [26, 32, 33, 44].

The abundance of *Bacteroidetes* (including *Parabacteroides* and *Bacteroides*) was reported to be lower in aGVHD patients than in non-GVHD patients [22, 38, 45]. A recent study showed that the increased abundance of *Bacteroides* in the intestinal microbiota was associated with improved GVHD, and administration of *Bacteroides fragilis* increased the diversity of the intestinal microbiota and significantly alleviated GVHD severity [46]. In contrast, one study in mice reported that *Bacteroides/Prevotella* spp. were expanded in GVHD [37].

The abundances of other bacteria, including *Ruminococcaceae*, *Eubacteriaceae*, *Peptostreptococcaceae*, *Actinomycetaceae* and *Faecalibacterium* spp. were also reported to be positively correlated with a reduced risk of GVHD and low GVHD-related mortality [21, 23, 35, 38]. Using shotgun metagenomic sequencing, Ilett et al. observed a lower abundance of *Akkermansia muciniphila* (*AKK*) in aGVHD patients, which was linked to an increased risk of aGVHD and suggested a protective role of *AKK* in aGVHD [33]. In contrast, a mouse model study showed that the *AKK* expansion induced by imipenem-cilastatin was associated with increased GVHD severity [47]. These contradictory results may be due to the different sequencing methods and species applied in the different studies.

Taken together, these findings demonstrate that alteration of the microbial composition is involved in the pathogenesis of aGVHD, and the abundances of several bacteria may be predictive biomarkers and therapeutic targets for aGVHD.

### Microbial metabolites and aGVHD

Microbial metabolites comprise diverse intermediate and end products produced by the intestinal microbiota and play critically important roles in regulating intestinal homeostasis and the immune response [48]. A shift in microbial metabolites caused by alteration of the microbial composition is reported to affect the development of aGVHD in allo-HSCT recipients.

Most studies reported that the levels of short-chain fatty acids (SCFAs), including acetate, butyrate, and propionate, were obviously reduced in subjects with aGVHD and associated with GVHD severity and mortality. One retrospective study on 316 allo-HSCT patients reported that the acetate, propionate and butyrate levels were reduced in severe aGVHD patients. Among these SCFAs, butyrate was dramatically reduced during all stages of acute GI GVHD and could serve as a diagnostic marker [32]. Propionate levels were shown to be significantly reduced post-HSCT and correlated with the progression of aGVHD in humans. The abundances of *Bacteroides* and *Parabacteroides* were associated with the level of propionate. Butyrate is the most studied SCFA in GVHD and lower fecal butyrate levels were observed in pediatric patients with aGVHD [38, 49]. Fecal butyrate levels were associated with the diversity of the intestinal microbiota after allo-HSCT. Furthermore, butyrate levels were positively correlated with the abundance of *Clostridiales* (including *Lachnospiraceae* and *Ruminococcaceae*) and negatively correlated with the abundances of *Enterococcus* and *Lactobacillus* [31]. Additionally, higher abundances of butyrate-producing bacteria reduced the risks of viral infection in the lower respiratory tracts of patients after allo-HSCT [50]. In another clinical study, patients with higher plasma levels of butyrate at study completion responded to treatment with urinary-derived human chorionic gonadotropin for steroid-refractory aGVHD compared to non-responders [51]. Butyrate concentrations are obviously decreased in the intestine after allo-HSCT in mouse models. Restoration of butyrate by administration of exogenous butyrate directly or feeding of 17 strains of high butyrate-producing *Clostridia* orally, can alleviate aGVHD [43]. Fujiwara et al. also reported that administration of butyrate and propionate mitigated aGVHD in a murine model, and butyrate treatment showed a good ability to protect against GVHD, whereas the administration of acetate had no effect on GVHD development. Notably, administration of higher doses of butyrate and propionate had no effect on GVHD development [52]. A randomized clinical trial studying the effect of resistant starch (RS, potato-starch) on aGVHD is ongoing (NCT02763033), and investigators hypothesized that RS is capable of increasing the butyrate levels in intestinal tissues and may thereby alleviate the progression of GVHD [53].

A recent study showed that the plasma levels of aryl hydrocarbon receptor (AhR) ligands, bile acids, polyamine metabolites and plasmalogens were significantly changed in subjects undergoing HSCT, especially in those with aGVHD. They observed significant decreases in the levels of microbiota-derived AhR ligands, including 3-indoxyl sulfate (3-IS), indoleacetate, indoleacetylglutamine, and indolepropionate in aGVHD patients [54]. Indoles and indole derivatives, as AhR ligands, are produced via tryptophan metabolism in microbes. High levels of indoles are associated with the expansion of *Bacteroidales*, *Lachnospiraceae* and *Akkermansia*, while low levels of indoles are associated with the expansion of *Enterococcus* and *Lactobacillus*. In addition to the correlation with microbial composition, indole levels are also positively correlated with microbial diversity [31]. Low levels of urine 3-IS are correlated with higher transplant-related mortality and lower overall survival and may be a potential predictive biomarker of GVHD [55, 56]. The abundances of *Lachnospiraceae* and *Ruminococcaceae* were associated with a higher level of 3-IS, whereas that of *Bacilli* was associated with a lower 3-IS level [55]. Similarly, another study revealed that indoxyl sulfate levels were reduced in the stool specimens of patients with aGVHD and negatively correlated with *Enterococci* and positively correlated with *Eubacterium rectale* and *Clostridium phytofermentalis* abundances [36]. In murine aGVHD models, administration of a commensal *E. coli* strain that delivered indole metabolites or administration of indole-3-carboxaldehyde (ICA) directly improved GVHD and reduced GVHD-related mortality while maintaining GVL effects [57].

A recent study revealed that administration of the microbial metabolite trimethylamine N-oxide (TMAO) increased the severity and mortality of aGVHD in mice. A high-choline diet that was metabolized into TMAO also exacerbated GVHD, whereas 3,3-dimethyl-1-butanol (DMB), a structural analog of choline, inhibited TMAO production and decreased TMAO-induced GVHD severity [58]. Significant alterations of other microbial metabolites, such as bile acids and polyamine metabolites, were observed in only aGVHD patients [54].

Collectively, these findings indicate that microbial metabolites play important roles in the development of aGVHD and may be potential therapeutic targets for the prevention and treatment of aGVHD; nevertheless, the mechanisms remain unclear and need to be further elucidated.

### Mechanisms of the intestinal microbiota in aGVHD

The role of the intestinal microbiota in the regulation of intestinal homeostasis and immune responses has been widely reported in inflammatory bowel disease, autoimmune disease and metabolic syndrome disease; however, fewer studies have focused on the mechanisms underlying its role in aGVHD. aGVHD is a complicated inflammatory process that is initiated by the activation of host antigen-presenting cells (APCs), which present antigens to donor T cells, followed by the activation of donor T cells to damage host tissues [59, 60]. Conditioning regimens (including irradiation, chemotherapy and antibiotics) for allo-HSCT can destroy the integrity of the intestinal barrier. Subsequently, intestinal bacteria and their components translocate into the intestinal lamina propria to regulate the progression of aGVHD [12]. The possible mechanisms by which microbiota regulate intestinal homeostasis and immune responses during aGVHD involve not only the microbiota itself (via microbial surface antigens) but also microbiota-derived metabolites.

### Intestinal barrier and aGVHD

The intestinal barrier is a dense structure composed of a monolayer of IECs, including classical epithelial cells, goblet cells, Paneth cells, enteroendocrine cells, enterocytes, M cells, and intestinal stem cells (ISCs), which exert diverse biological functions [61].

Goblet cells secrete mucin to form a mucus layer as a physical barrier to separate the intestine from luminal contents [62]. Administration of IL-25 promotes the expansion of goblet cells through Lypd8, thereby ameliorating GVHD in a mouse model [63]. Paneth cells, which reside in crypts, are the target of GVHD, defend against pathogens and regulate immune responses through the production of antimicrobial peptides (AMPs), including defensins and C-type lectin regenerating islet derived protein 3 (REG3; REG3α in humans and REG3γ in mice) [61, 64, 65]. The loss of Paneth cells and the reduction in α-defensin levels are correlated with GVHD exacerbation in humans and mice, which indicates that Paneth cells may serve as a biomarker of GI aGVHD [22, 66]. Consistently, fecal α-defensin levels are significantly decreased in mice with GVHD and associated with intestinal microbial diversity [67]. REG3α has been demonstrated to be a plasma biomarker of GI GVHD [68]. The levels of REG3α/γ are decreased during aGVHD, concomitant with reduced numbers of ISCs and Paneth cells. Administration of IL-22 can repair the integrity of the intestinal barrier and ameliorate GVHD by regulating the secretion of REG3γ from Paneth cells [69]. Loss of enteroendocrine L-cells and reduced levels of glucagon-like peptide-2 (GLP-2) secreted by L-cells are observed in aGVHD and correlated with its clinical severity and patient outcomes. Treatment with teduglutide, a GLP-2 analog, changes the microbial composition and restores intestinal homeostasis by promoting the regeneration of Paneth cells and ISCs, thereby alleviating aGVHD while preserving the GVL effects in mice [70]. ISCs can differentiate into all subsets of IECs, thereby maintaining intestinal homeostasis. After allo-HSCT, donor T cells can translocate to the ISC compartment and directly damage ISCs [71]. Injection of the Wnt agonist R-spondin1 (R-Spo1) promotes the proliferation of ISCs through the Wnt signaling pathway to alleviate GVHD [72]. R-Spo1 can also induce ISCs to differentiate into Paneth cells and increase the secretion of α-defensins which prevent intestinal microbiota dysbiosis and maintain intestinal homeostasis [73]. An inhibitor of the stress protein HSP90 (17AAG) improves aGVHD by increasing the numbers of ISCs and Paneth cells, as well as the expression of defensins [74]. In a mouse aGVHD model, maintaining intestinal barrier integrity prevents the dysbiosis of intestinal microbial diversity and mitigates aGVHD [75]. Taken together, these studies indicate that the intestinal barrier plays a critical role in the pathophysiology of aGVHD.

Butyrate, a microbial metabolite mentioned above, was previously shown to repair the intestinal barrier integrity and inhibit IEC apoptosis, potentially by decreasing the levels of histone acetylation in IECs, thereby alleviating aGVHD [43]. In a subsequent study, the Reddy group further elucidated that SCFAs (butyrate and propionate) were able to bind G protein-coupled receptor 43 (GPR43) on IECs to promote ERK phosphorylation through activation of the NLRP3 inflammasome, which was critical for reducing aGVHD severity [52]. These findings indicate that the intestinal microbiota is important for maintaining the intestinal barrier integrity and homeostasis.

### Intestinal immune response and aGVHD

The crosstalk between the intestinal microbiota and immune cells is key to maintaining intestinal homeostasis. A recent large-scale study reported a link between the intestinal microbiota and the dynamics of immune cells in allo-HSCT patients [76]. The Han group demonstrated that decreased abundances of *Lachnospiraceae* and *Ruminococcaceae* and an increased level of *Enterobacteriaceae* were associated with an imbalance of Treg/Th17 cells, possibly through acetylated H3 in CD4^+^ T cells in aGVHD patients [29]. Moreover, the balance of Treg/Th17 cells and the levels of inflammatory factors were associated with aGVHD [21]. These findings indicate that the microbiota can modulate the immune response during aGVHD.

Following destruction of the intestinal barrier, bacteria and microbial metabolites translocate into the intestinal lamina propria to regulate the immune response. Microbiota-associated molecular patterns can be recognized by pattern recognition receptors (PPRs), including Toll-like receptors (TLRs) and NOD-like receptors (NLRs), expressed on APCs, neutrophils and IECs. This process induces the activation of APCs, resulting in the activation and expansion of donor T cells and further causing a systemic inflammatory response to promote the progression of aGVHD [12].

TLRs play an important role in the occurrence and severity of aGVHD [77]. TLR4 is the receptor of lipopolysaccharide (LPS), the major outer membrane component of gram-negative bacteria, which is essential for aGVHD initiation. Antagonism of LPS infusion reduces GVHD in mice [78]. Mutations in TLR-4 on the donor/patient side increase the risk of severe and GI aGVHD in patients [79], while mutations in TLR4 in patients reduce the risk of GVHD [80]. TLR-4 depletion in either donors or recipients alleviates aGVHD in mice [81]. Conversely, another study in mice showed that the increased expression of TLR-4 in intestinal tissues might play a protective role in aGVHD by increasing the production of tissue-protective factors and inhibiting IEC apoptosis [82]. Therefore, TLR4 plays a dual role in the pathogenesis of GVHD, possibly due to its differential expression in different cell types in the host and donor. Flagellin, a TLR-5 agonist extracted from bacterial flagella, reduces GVHD by suppressing the early activation of donor T cells and reducing proinflammatory cytokine production [83]. A TLR7/8 agonist, 3 M-011, significantly ameliorates aGVHD by inducing indoleamine 2,3-dioxygenase production and mitigating intestinal injury in mice [84]. Heimessat et al. demonstrated that bacterial sensing via TLRs was essential for the development of GVHD in a mouse model, and depletion of TLR9 could reduce mortality [37]. Correspondingly, CpG oligodeoxynucleotides (ODNs) were found to aggravate GVHD by activating the secretion of IFNγ from APCs via recognition by TLR9 [85]. Similar to TLRs, NLRs are important in aGVHD. NOD2 mutations on the donor side reduce the risk of severe aGVHD, whereas NOD2 gene mutations on the patient and donor sides increase the risk of severe aGVHD after transplantation [79]. NOD2 deficiency in recipient mice exacerbates GVHD [86].

After translocation of the intestinal microbiota, neutrophils can be recruited to the intestinal tract and impair intestinal tissues via reactive oxygen species, thereby aggravating aGVHD. Depletion of neutrophils reduces GVHD-related mortality [87]. In addition, neutrophils can migrate to mesenteric lymph nodes from the ileum after disruption of the intestinal barrier and present alloantigens to T cells, resulting in the activation and expansion of alloreactive T cells, thereby increasing the severity of aGVHD [88]. The shift in the intestinal microbiota plays an important role in the regulation of the proinflammatory effects of neutrophils. The activation of neutrophils by translocating bacteria is attenuated by a lack of TLR expression [87]. Moreover, treatment with an antibody specific for poly-N-acetylglucosamine, which is expressed on various pathogens, reduces the proliferation of neutrophils, thereby decreasing the mortality of mice with aGVHD [89]. In addition to modulation of the neutrophil-mediated immune response, the intestinal microbiota also regulates the immune function of macrophages. *Enterococcus* reportedly promotes the development of aGVHD by directly impairing intestinal epithelial tissues through toxin-mediated intestinal inflammation and damage as well as by inducing the release of TNF-α from macrophages [66]. The microbial metabolite TMAO has been shown to promote the polarization of M1 macrophages via NLRP3 inflammasome activation. The increased expansion and infiltration of M1 macrophages in target organs induce Th1 and Th17 cells, which exacerbates GVHD in mice [58].

IL-22-producing innate lymphoid cells (ILCs) play an important role in maintaining intestinal homeostasis, and the number of ILCs and level of IL-22 are reportedly reduced during the progression of aGVHD. Consistently, the activation of ILCs is associated with a decreased risk of aGVHD. Moreover, depletion of IL-22 in the recipient severely damages intestinal epithelial integrity and consequently leads to aGVHD aggravation and increased mortality [90, 91]. An additional study revealed that IL-22 promoted the proliferation of ISCs by inducing the phosphorylation of STAT3 in Lgr5^+^ ISCs [92]. Indole-3-aldehyde, an indole derivative produced by *Lactobacilli*, upregulates the production of IL-22 by activating AhRs on ILCs, which induces the production of REG3γ from Paneth cells [93–95]. In consideration of these findings, a phase II clinical trial on IL-22 IgG2-Fc (F-652) in combination with systemic corticosteroids for the treatment of GI aGVHD has been conducted, but the results have not been reported until now (NCT02406651). In addition, another indole derivative, ICA, acts via type I interferon signaling to protect against and repair intestinal barrier damage, subsequently improving aGVHD [57].

The rapid reconstitution of NK and B cells is positively correlated with the high abundances of the *Ruminococcaceae* and *Lachnospiraceae* families in patients with no or mild aGVHD, indicating that intestinal microbiota might also regulate NK and B cell immune responses [35]. The development of the B cell lineage is influenced by the microbiota colonization in the intestinal lamina propria [96]. B cells in the intestinal lamina propria secrete soluble IgA to regulate the interactions between the host and microbiota and ensure the compartmentalization of commensal bacteria from the intestinal epithelium [97].

Motoko et al. demonstrated that the microbiota affected the expression of major histocompatibility (MHC) class II molecules on IECs via the IL-12/IFNγ cytokine axis under both healthy and inflammatory conditions. The upregulated expression of MHC class II molecules on IECs was shown to activate CD4^+^ T cells and initiate lethal GVHD through alloantigen presentation. IL-12/23p40 neutralization was shown to inhibit the increased expression of MHC class II molecules on IECs and prevent the initiation of lethal GVHD [98]. *Bacteroides fragilis* can alleviate GVHD by inhibiting T cell activation and proliferation in a polysaccharide A (PSA)-dependent manner. *Bacteroides fragilis* also preserves intestinal integrity possibly by regulating SCFAs, IL-22 and Tregs, thereby improving GVHD. It is worth noting that the administration of *Bacteroides fragilis* does not affect GVL activity [46]. In a mouse aGVHD model, butyrate mitigates GVHD by decreasing the intestinal infiltration of donor CD4^+^ and CD8^+^ T cells and inhibiting their activation [43]. Moreover, butyrate does not affect the number of Tregs despite that it has been shown to induce Tregs to downregulate the inflammatory response [99, 100].

Mucosal-associated invariant T (MAIT) cells are a unique innate-like T cell subset that can recognize riboflavin metabolites from distinct bacteria and fungi. The reconstitution of MAIT cells is also positively correlated with the abundance of *Blautia* spp. in the intestine. Consistently, lower numbers of MAIT cells are associated with severe aGVHD [101]. Recipient MAIT cells mitigate aGVHD in mice by regulating the intestinal barrier integrity and microbial diversity, and decreasing the numbers of proinflammatory donor Th1 and Th17 cells in the colon. MAIT cells also produce IL-17A, and recipients deficient in IL-17A aggravate GVHD [102]. Another study found that recipient-derived IL-17 mitigated aGVHD through IL-17 RA signaling. The transfer of IL-17RA^−/−^ mouse microbiota into WT mice significantly aggravated aGVHD, indicating the involvement of IL-17-sensitive microbiota [103]. These results indirectly suggest that MAIT cells may affect the development of aGVHD by producing IL-17A to influence microbial composition.

**Fig. 2:**
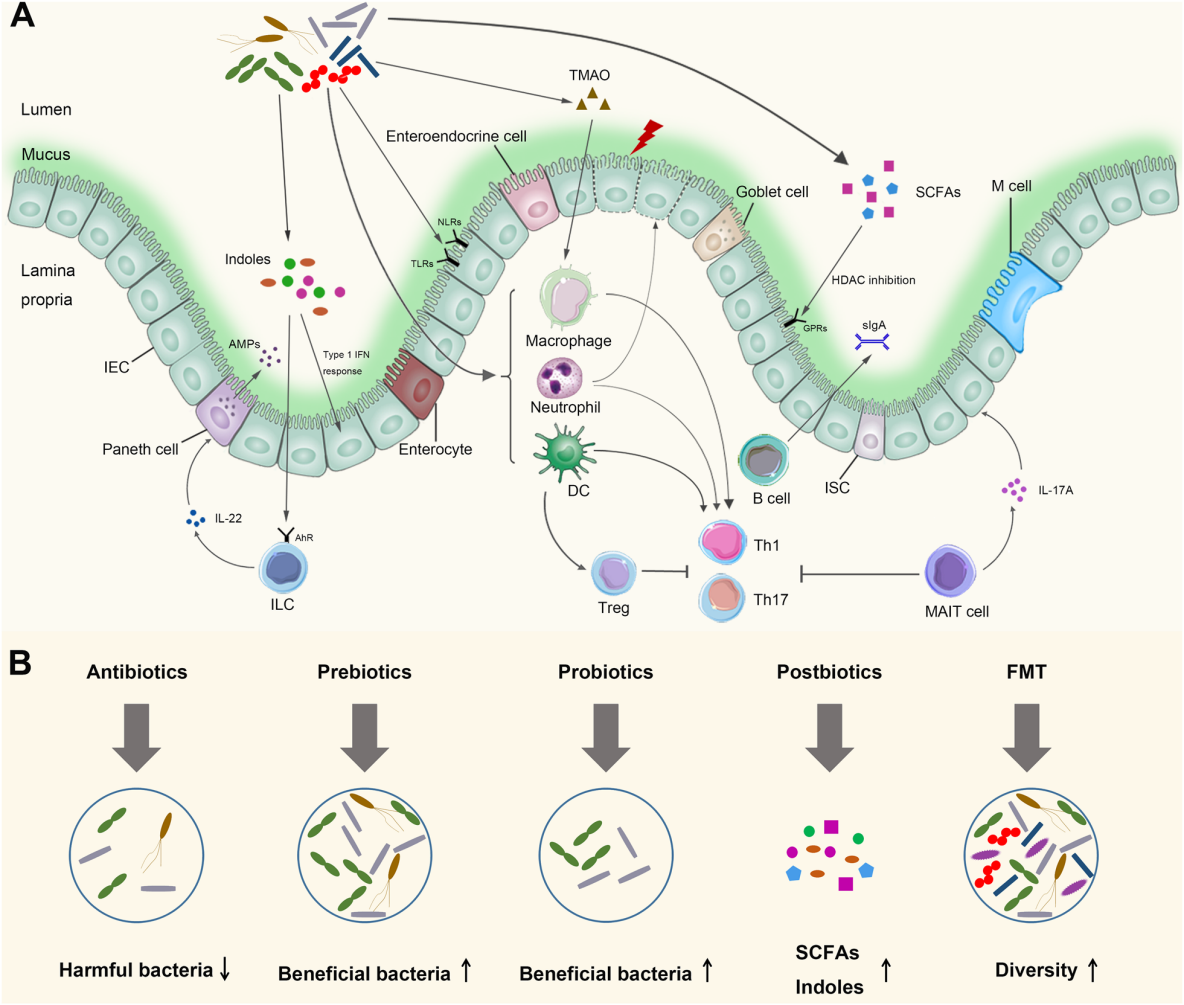
Modulation mechanisms of the intestinal microbiota during GVHD and potential solutions of microbiota dysbiosis. **A** During allo-HSCT, conditioning regimens (including total body irradiation, chemotherapy and antibiotics) disrupt integrity of intestinal barrier. Intestinal bacteria and their metabolites translocate into the intestinal lamina propria to regulate the progression of aGVHD. Microbiota-associated molecular patterns can be recognized by the PPRs expressed on APCs, neutrophils and IECs, including TLRs and NLRs. APCs regulate the activation and expansion of donor T cells, including Treg, Th1 and Th17 cells. Neutrophils can aggravate aGVHD through impairing the intestinal tissues via reactive oxygen species. B cells in the intestinal lamina propria secrete soluble IgA to regulate the interactions between the host and microbiota, and development of B-cell lineage is influenced by the intestinal microbiota. Recipient MAIT cells mitigate aGVHD by maintaining intestinal barrier integrity and decreasing the proinflammatory donor Th1 and Th17 cells. MAIT cells also produce IL-17A to alleviate aGHVD. Indoles improve aGVHD via type I interferon response or activating AhRs on ILCs, which protect and repair the intestinal barrier from damage. SCFAs repair intestinal barrier integrity and inhibit IECs apoptosis, resulting in alleviating aGVHD. TMAO promotes the polarization of M1 macrophage which induces the activation of Th1 and Th17 cells, resulting in GVHD exacerbation. IEC, intestinal epithelial cell; ISC, intestinal stem cell; AMPs, antimicrobial peptides; SCFAs, short chain fatty acids; TMAO, trimethylamine N-oxide; ILC, innate lymphoid cell; MAIT, mucosal-associated invariant T cell; Th1, T helper 1 cell; Th17, T helper 17 cell; Treg, regulatory T cell; TLR, Toll-like receptor; NLRs, NOD-like receptors; AhR, aryl hydrocarbon receptor; GPR, G-protein-coupled receptor. **B** Interventions strategies modulating intestinal microbiota mainly include antibiotics, prebiotics, probiotics, postbiotics and FMT

In summary, the intestinal microbiota and its metabolites can impact the immune system directly or indirectly to regulate the progression of aGVHD (summarized in Fig. 2); however, the in-depth mechanisms of most intestinal microbiota and metabolites need to be further clarified.

### Interventions targeting the intestinal microbiota in aGVHD

Previous studies have demonstrated the importance of the diversity and composition of the intestinal microbiota in subjects with aGVHD. Several specific intestinal bacteria, such as *Clostridiales* and *Bacteroides*, have been shown to play protective roles in aGVHD. Therefore, regulating intestinal microbiota homeostasis and maintaining the dominance of beneficial bacteria is a feasible way to prevent aGVHD in allo-HSCT patients. Currently, strategies to modulate the intestinal microbiota mainly include antibiotics, prebiotics, probiotics, postbiotics and FMT (Fig. 2). Tables 2 and 3 summarize the published and main ongoing studies related to microbiota interventions for the treatment and prevention of aGVHD, respectively.

**Table 2:** The published clinical trials of microbiota intervention in the treatment and prevention of aGVHD

Interventions	Number of patients	Outcomes	References
**Antibiotics**			
Optimization of Antibiotic	140 HSCT recipients including intervention cohort and control cohort	The first prospective multicentre study aims to address the effect of two antimicrobial therapy strategies on intestinal microbiota. Faecal microbiota and clinical outcomes including GVHD incidence and severity will be compared between both cohorts.	[104]
**Prebiotics**			
RS and GFO	Prebiotic group: 49 Control group: 142	RS and GFO administration mitigated mucosal injury and reduced the incidence of aGVHD. RS and GFO intake maintained the gut microbial diversity and preserved butyrate-producing bacterial population	[105]
FOS	Prebiotic group: 15 Control group: 16	FOS was safe and well-tolerated at 10 g per day in allo-HSCT patients. Community-level gut microbial composition was only different on transplant day (day 0) between FOS and controls, 5 days after intake of FOS. The incidence of aGVHD (grades I-IV) and overall survival at 1 year were not associated with the administration of FOS	[106]
Glutamine	Prebiotic group: 27 Control group: 26	Glutamine administration decreased GVHD related mortality	[107]
**Probiotics**			
*Lactobacillus Rhamnosus GG*	Probiotic group: 20 Control group: 11	The abundance of *Lactobacillaceae* family and *Lactobacillus* genus were not affected by probiotic administration. The administration of *Lactobacillus Rhamnosus GG* did not affect the gut microbiota or the incidence of aGVHD	[108]
*Lactobacillus plantarum* strains 299 and 299v	Probiotic group: 30	Administration of *Lactobacillus plantarum* was safe and feasible in children and adolescents undergoing HCT	[109]
**FMT**			
Related or spouse donor	4 patients with GI aGVHD (SR, n = 3; steroid-dependent, n = 1)	FMT was safe and effective in all patients, with 3 CR and 1 PR. The dynamic of microbiota seemed to be linked to the gut condition of the patients. FMT also increased the peripheral effector regulatory T cells	[110]
Unrelated or related donor	3 patients with SR GI aGVHD	All three patients responded clinically to FMT, with 2 CR and 1 PR	[111]
Unrelated donor	8 patients with SR GI aGVHD	FMT was safe and effective in most patients, with 4 CR, 1 PR and 1 remission	[112]
Related donor	1 patient with SR GI aGVHD	FMT restored the intestinal microbial diversity, with the improvement of diarrhea and colonoscopy findings	[113]
Unrelated donor	2 patients with SR GI aGVHD	FMT resulted in 1CR and 1 PR	[114]
Unrelated donor	1 patient with SR GI aGVHD	The diversity and structure of the intestinal microbiota after FMT was restored and the patients was cured	[115]
Unrelated donor	15 patients with GI aGVHD (SR, n = 6; steroid-dependent, n = 9)	10 patients showed CR, accompanied by an increase in intestinal microbial diversity and increased abundance of butyrate-producing bacteria	[116]
Unrelated donor	41 patients with SR GI GVHD FMT group: 23 Control group:18	Clinical remission was significantly greater in FMT group than in control group. FMT group showed a higher overall survival and lower mortality rate. No differences were observed in the occurrence of any other side effects	[117]
Unrelated donor	11 patients with aGvHD	FMT was effective in the treatment of GVHD and in the decolonization of GI tract from antibiotic-resistant bacteria	[118]

RS, resistant starch; GFO, glutamine, fiber, and oligosaccharides; SR GI aGVHD, steroid-resistant gastrointestinal acute GVHD; CR, complete responses; PR, partial response

**Table 3:** Ongoing clinical trials of microbiota intervention in the treatment and prevention of aGVHD

Study title	Interventions	Phases	NCT Number
**Antibiotics**			
Optimization of Antibiotic Treatment in Hematopoietic Stem Cell Receptors	Procedure: Optimization cohort; Procedure: Control cohort	N/A	NCT03727113
Gut Decontamination In Pediatric Allogeneic Hematopoietic	Drug: Vancomycin-polymyxin B	Phase 2	NCT02641236
Human Lysozyme Goat Milk for the Prevention of Graft Versus Host Disease in Patients With Blood Cancer Undergoing a Donor Stem Cell Transplant	Drug: Cyclophosphamide; Drug: Etoposide; Drug: Goat Milk; Biological: Palifermin; Drug: Sirolimus; Drug: Tacrolimus	Phase 1	NCT04177004
**Prebiotics**			
Dietary Manipulation of the Microbiome-metabolomic Axis for Mitigating GVHD in Allo HCT Patient	Drug: potato-starch; Other: Starch Placebo	Phase 2	NCT02763033
The Use of A Prebiotic to Promote a Healthy Gut Microbiome in Pediatric Stem Cell Transplant Recipients	Drug: Inulin; Drug: Placebos	N/A	NCT04111471
Oral Supplementation of 2'-Fucosyllactose in Allogeneic Bone Marrow Transplant Recipients	Drug: 2'-fucosyllactose; Other: Placebo (2 g oral glucose)	Phase 1/Phase 2	NCT04263597
Prebiotic Galacto-oligosaccharide and Acute GVHD	Dietary Supplement: Galacto-oligosaccharide; Dietary Supplement: Maltodextrin	Phase 1/Phase 2	NCT04373057
Effects of Prebiotics on Gut Microbiome in Patients Undergoing HSCT	Other: Pre-biotic foods/drinks	N/A	NCT04629430
High Dose Vitamin A in Preventing Gastrointestinal GVHD in Participants Undergoing Donor Stem Cell Transplant	Dietary Supplement: Vitamin A Compound	N/A	NCT03719092
**Probiotics**			
*Lactobacillus Plantarum* in Preventing Acute Graft Versus Host Disease in Children Undergoing Donor Stem Cell Transplant	Biological: *Lactobacillus plantarum strain 299*; Biological: *Lactobacillus plantarum strain 299v*; Other: Placebo Administration	Phase 3	NCT03057054
CBM588 in Improving Clinical Outcomes in Patients Who Have Undergone Donor Hematopoietic Stem Cell Transplant	Drug: Clostridium butyricum CBM 588 Probiotic Strain; Other: Best Practice	Phase 1	NCT03922035
MaaT013 as Salvage Therapy in Ruxolitinib Refractory GI-aGVHD Patients	Drug: MaaT013, which is made of allogeneic, full-ecosystem pooled biotherapeutic intestinal microbiota	Phase 3	NCT04769895
**FMT**			
Prospective Study of FMT for Acute Intestinal GVHD After Allo-HSCT	Biological: Fecal Microbiota Transplantation; Drug: drug	N/A	NCT04711967
Fecal Microbiota Transplantation For The Treatment Of Gastro-Intestinal Acute GVHD	Biological: Fecal Microbiota Transplantation	Phase 2	NCT04059757
FMT In High-Risk Acute GVHD After ALLO HCT	Biological: Fecal Microbiota Transplant	Phase 1	NCT04139577
Fecal Microbiota Transplantation With Ruxolitinib and Steroids as an Upfront Treatment of Severe Acute Intestinal GVHD	Biological: allogeneic fecal microbiota; Drug: Ruxolitinib; Drug: Methylprednisone	Phase 1/Phase 2	NCT04269850
Fecal Microbiota Transplantation for the Treatment of Severe Acute Gut Graft-Versus-Host Disease	Drug: Fecal Microbiota Transplantation Capsule	Phase 1	NCT04280471
FMT for Steroid Resistant Gut Acute GVHD	Biological: Fecal Microbiota Transplantation	Early Phase 1	NCT04285424
Efficacy and Safety of FMT Capsule Treating Steroid-refractory GI-aGvHD	Biological: Fecal Microbiota Transplant Capsule	N/A	NCT04622475
Fecal Microbiota Transplantation in aGvHD After ASCT	Biological: Fecal microbiota transplantation	Phase 3	NCT03819803
Efficacy and Safety of Auto-FMT in Preventing aGVHD	Other: autologous fecal bacteria	Phase 1	NCT04745221
Fecal Microbiota Transplantation in Gut aGVHD Treated	Biological: Fecal microbiota	None	NCT03148743
Fecal Microbiota Transplantation for Steroid Resistant/Dependent Acute GI GVHD	Biological: fecal microbiome transplantation	Phase 2	NCT03812705
Fecal Microbiota Transplantation for Treatment of Refractory Graft Versus Host Disease-a Pilot Study	Biological: Fecal Microbiota Transplantation	Phase 1	NCT03549676
Safety and Efficacy of Fecal Microbiota Transplantation	Procedure: Fecal Microbiota Transplantation	N/A	NCT04014413
**Others**			
Randomized, Prospective, Multicenter Study to Compare Enteral Nutrition to Parenteral Nutrition as Feeding Support in Patients Presenting Malignant Hemopathy Who Underwent an Allogeneic Hematopoietic Stem Cell Transplantation	Drug: Enteral nutrition alanyl-glutamin, Dipeptiven	Phase 3	NCT01955772

### Antibiotics

Antibiotics are commonly used in allo-HSCT patients. As mentioned above, germ-free mice showed a reduced risk of aGVHD and improved aGVHD, and preclinical studies indicated that the intestinal microbiota played a critical role in the pathogenesis of aGVHD. Accordingly, gut decontamination with non-absorbable antibiotics was used in allo-HSCT patients. Nonetheless, accumulating evidence shows that antibiotics must be carefully selected (summarized in Table 4), as the composition and diversity of intestinal microbiota shaped by antibiotic treatment may lead to different clinical outcomes, either increasing or decreasing the GVHD risk.

**Table 4:** Impact of the type of antibiotics on aGVHD

Antibiotics	Outcomes	References
Imipenem-cilastatin	Treatment with imipenem-cilastatin was associated with the high risk of GVHD-related mortality, incidence of grades II–IV GVHD and GI GVHD	[47]
Penicillins including penicillin, penicillin derivatives and piperacillin-tazobactam	Penicillins and its derivatives were associations with increased risk of aGVHD. Exposure to piperacillin-tazobactam increased the incidence of grade II–IV GVHD and GI GVHD, and increased GVHD-related mortality	[47, 119–121]
Carbapenems	Carbapenems were associated with the increased risk of grade II–IV aGVHD and intestinal GVHD. Early and longer use of carbapenem especially increased aGVHD risk	[119–123]
Fourth-generation cephalosporins	The cumulative incidence of GI aGVHD was significantly higher in patients who received fourth-generation cephalosporins than in those who did not	[124]
Cephalosporins	There was no association between cephalosporins treatment and aGVHD incidence	[119]
Glycopeptide	Patients with GI GVHD received significantly longer administration of glycopeptide compared to those without GI GVHD	[122]
Aminoglycosides	There was no association between aminoglycosides treatment and aGVHD incidence	[124]
Quinolones	There was no association between quinolones treatment and aGVHD incidence	[122, 124]
Aztreonam	Treatment with aztreonam was associated with a decreased GVHD-related mortality by univariate analyses	[47]
Cefepime	Antibiotic exposure to cefepime was significantly correlated with reduced GVHD-related mortality by univariate analyses	[47]
Rifaximin	Patients received rifaximin showed lower transplant-related mortality and higher overall survival	[56, 125]

Generally, treatment with broad-spectrum antibiotics causes a dramatic shift in the intestinal microbial composition and is associated with a higher incidence of aGVHD in adult and pediatric patients [47, 119, 122–124, 126, 127]. At 5 years post-transplantation, GVHD-related mortality was increased in 857 allo-HSCT recipients treated with imipenem-cilastatin or piperacillin-tazobactam antibiotics [47]. In another study on 399 patients, sequential exposure to penicillin derivatives and carbapenems was regarded as an independent risk factor for GI aGVHD [120]. The administration of fourth-generation cephalosporins was shown to significantly increase the risk of aGVHD [124]. A recent study showed that anaerobic antibiotics, piperacillin-tazobactam and carbapenems, were positively correlated with a higher risk of aGVHD and higher GVHD-related mortality [121].

Consistently, antibiotics with a narrow spectrum, such as rifaximin, cefepime and aztreonam, are associated with reduced GVHD severity [47, 56, 121, 125]. Furthermore, some narrow-spectrum antibiotics lead to the domination of harmful bacteria or loss of beneficial bacteria in the gut. Vancomycin and metronidazole are associated with the abundance of *Enterococcus*, which has been proven to play a pathogenic role in aGVHD [40, 128].

Finally, the outcomes of allo-HSCT patients treated with antibiotics pre-transplant were worse than those of patients treated with antibiotics after the transplant [127]. Therefore, it should be noted that the proper selection and timing of antibiotic administration must be carefully determined according to the patient’s situation. Current clinical investigations suggest that the short-term application of antibiotics with a narrow spectrum can eliminate harmful bacteria while retaining beneficial bacteria. Additionally, intestinal bacterial antagonism has been shown to protect organisms from bacterial infection [129, 130]. Moreover, human lysozyme acts as an antimicrobial agent, as it supports the growth of beneficial gut bacteria and reduces the growth of harmful bacteria. A randomized pilot study on human lysozyme goat milk in preventing GVHD in patients with blood cancer undergoing donor stem cell transplantation is underway (NCT04177004). Based on the type VI secretion system and surface-displayed nanobodies that mediate antigen-specific cell–cell adhesion, Ting et al. developed programmed inhibitor cells that deplete target gram-negative bacteria in a complex bacterial community, which has potential as a novel antibiotic strategy [131].

### Prebiotics

Prebiotic treatment involves the administration of some special foods or food components, such as carbohydrates and fibers that are metabolized by the microbiota but cannot be digested by humans, to support the competitive advantages of beneficial bacteria. Alternatively, the administration of prebiotics may augment the production of beneficial metabolites, including SCFAs, through bacterial fermentation, thereby improving aGVHD by maintaining intestinal integrity and regulating the immune response [43, 52]. A previous prebiotic study showed that nutritional supplementation comprising glutamine, fiber, and oligosaccharides (GFO) alleviated mucosal injury after allo-HSCT [132]. Another clinical study revealed that GFO and RS reduced the overall aGVHD risk and decreased the incidence of grade II-IV aGVHD. Notably, butyrate-producing bacteria and fecal butyrate concentrations were well maintained or increased after GFO and RS treatment [105]. Administration of glutamine restores intestinal integrity, reduces inflammation and mitigates aGVHD in mice [133, 134]. Oral glutamine treatment also reduces mucositis and GVHD in patients [135]. Lower levels of vitamin A increase the incidence of GI GVHD possibly by increasing intestinal permeability, indicating that supplementation with vitamin A may improve the outcomes of allo-HSCT patients [136]. In clinical trials on allo-HSCT patients, several prebiotic strategies have been investigated, including RS (potato starch), inulin, fructo-oligosaccharides, human milk oligosaccharides (2’-fucosyllactose), galacto-oligosaccharides and vitamin A (Table 3). Notably, Yoshifuji et al. demonstrated that a low microbial diversity before allo-HSCT might dampen the beneficial effect of prebiotics [105]. Therefore, the timing of prebiotic treatment may be important for patient benefits, and further evaluation is needed.

### Probiotics

A probiotic strategy is defined as the direct supplementation of select live microbes that have health benefits, including single bacterial strains and strain mixtures. Decreased intestinal microbial diversity or domination of harmful bacteria is consistently found in the intestinal microenvironment of patients with GVHD, as mentioned above. Administration of beneficial bacteria is feasible to reconstitute the intestinal bacterial community. A pilot trial proved that the oral probiotic *Lactobacillus plantarum* is safe and feasible in children and adolescents undergoing HSCT, and most recipients have no bacteremia or adverse events [109]. Currently, a randomized phase III trial is ongoing to study the effect of *Lactobacillus plantarum*a on preventing aGVHD in children after allo-HSCT (NCT03057054). Previous studies have reported that the abundance of *Clostridiales* is negatively correlated with the risk of aGVHD in patients, and the administration of high butyrate-producing *Clostridia* alleviates aGVHD [42, 43]. A randomized open-label pilot study on *Clostridium butyricum MIYAIRI 588* (CBM588) in recipients of allo-HSCT is underway (NCT03922035). In a phase II trial, administration of *Lactobacillus brevis* CD2 lozenges prevented oral mucositis in HSCT patients undergoing high-dose chemotherapy [137]. Supplementation with *Lactobacillus rhamnosus GG* has been demonstrated to improve survival and reduce aGVHD severity in a mouse model [138]. Nonetheless, one clinical trial on 21 patients receiving probiotics and 10 patients receiving no intervention as a control did not reveal probiotic-related associations or changes in the incidence of GVHD [108]. One possible explanation is that humans feature person-specific gut mucosal colonization against transient probiotic colonization, thereby concealing the potential efficacy of probiotic therapy [139]. Additionally, common probiotic organisms may cause bloodstream infection in immunocompromised patients [140]. Thus, future efforts related to precision medicine considering personal intestinal homeostasis and immune capacity are required to ensure the safety and efficiency of probiotics in patients undergoing HSCT.

### Postbiotics

Postbiotics refer to beneficial molecules or metabolic products from probiotic bacteria that promote intestinal homeostasis [141]. Compared to live microorganisms, postbiotics lack microbe-associated molecular patterns (MAMPs) that potentially activate innate immunity and inflammation [142]. This strategy is safer than the probiotic strategy, without causing a risk of bacteremia in immunocompromised patients. To date, various beneficial effects of postbiotics, including maintaining the GI tract barrier surface, modulating intestinal epithelial cell damage, and modulating innate and adaptive host immune responses, have been demonstrated both in vitro and in murine models. Two SCFAs, butyrate and propionate, were shown to directly protect IECs and reduce aGVHD severity in mice [43, 143]. Microbiota-derived AhR ligands, including indoles and their derivatives, were shown to markedly alleviate aGVHD in a mouse model [57]. RS, which increases butyrate production, is being used as a prebiotic in an ongoing clinical trial for the prevention of aGVHD (NCT02763033), which suggests that butyrate has potential as a postbiotic therapeutics for aGVHD. However, the postbiotic strategy has not been utilized in HSCT patients.

### FMT

FMT refers to the transplantation of sterile fecal filtrates from healthy donors to the GI tracts of recipient patients. The efficacy of FMT was first demonstrated in patients with recurrent *Clostridium* difficile infection (rCDI). The mechanism of FMT in treating rCDI is related to the reconstruction of intestinal microbial diversity and the regulation of the immune response. Several subsequent trials also provided convincing evidence that FMT contributed to intestinal homeostasis. Therefore, FMT has been listed in clinical practice guidelines [144–146]. In the scenario of aGVHD prevention and treatment, several independent studies performed by different centers reported the efficiency of FMT in restoring intestinal diversity and beneficial bacteria. Kakihana et al. first conducted a pilot study to evaluate the safety of FMT from related donors in GI aGVHD patients (steroid-resistant, n = 3; steroid-dependent, n = 1). FMT was safe and effective in these patients, and no adverse events were reported, indicating that FMT has potential as a therapeutics for preventing aGVHD [110]. Two clinical studies conducted by our group reported that FMT from unrelated donors improved steroid-resistant GI GVHD and prolonged the survival time. This beneficial effect of FMT was associated with the restoration of microbial diversity [112, 117]. Other clinical studies also demonstrated that FMT from related or unrelated donors was effective for the treatment of aGVHD, especially steroid-resistant GI aGVHD [111, 113–116, 147]. A newly published study revealed that FMT was effective for the treatment of GVHD patients by promoting gut decolonization from antibiotic-resistant bacteria [118]. Currently, many clinical studies are investigating the effect of FMT on aGVHD (Tables 2 and 3).

Nonetheless, the expanding use of FMT for the treatment of CDI, inflammatory bowel disease, and irritable bowel syndrome has revealed its side effects, which include abdominal discomfort, bloating, transient low-grade fever, flatulence high-grade fever, infection and sepsis or even induction of chronic disease [148]. Furthermore, DeFilipp et al. reported one case of death after FMT, which was caused by the transmittance of donor drug-resistant *Escherichia coli* to the recipient [149]. Therefore, large-sample clinical studies are needed to confirm the efficacy and safety of FMT in patients undergoing allo-HSCT.

These reported adverse effects highlight the need for donor screening and careful benefit-risk assessment when designing FMT studies. The source of FMT can be related donors, unrelated donors or the recipients themselves. Related donors may have intestinal microbial composition similar to those of recipients. Autologous FMT (auto-FMT) may be safer than heterologous FMT due to the lower risk of potential pathogens from the autologous donor [150]. A study demonstrated that auto-FMT reestablished intestinal microbial diversity following allo-HSCT [151], and this randomized controlled clinical trial is still ongoing (NCT02269150). A clinical study on the efficacy and safety of auto-FMT in preventing aGVHD is currently being conducted by our group (NCT04745221). However, FMT from related donors and auto-FMT require a substantial amount of preparation time, ranging from sample collection to sterile fecal filtrate acquisition, while unrelated healthy donor fecal material can be obtained from a stool bank, which may be useful for large-scale and flexible applications [150]. DeFilipp and his colleagues showed that third-party FMT expanded microbial diversity and was feasible and safe after allo-HSCT [152]. Therefore, the screening of FMT donors is an important element to minimize adverse events and may be adjusted to the patient’s specific requirements.

Additionally, FMT can be administered via several modes, including colonoscopy, enema, esophagogastroduodenoscopy via enteric tubes (nasogastric, nasoduodenal, and nasojejunal tubes), and oral capsules [153], which still need to be investigated and further optimized. Moreover, the gut bacteriomes, mycobiomes and viromes of teenagers with GVHD after FMT displayed distinct compositional alterations and variable temporal dynamics, suggesting that bacterial, fungal and viral communities respond differently to FMT [154]. Randomized control trials on the impact of FMT on fungal and viral communities in large cohorts in the context of GVHD are needed for further evaluation.

### Others

Furthermore, the types of nutritional support, including enteral nutrition (EN) and parenteral nutrition (PN), also modulate the intestinal microbiota. EN provides nutrients to the gastrointestinal tract via a tube, while PN provides nutrients to the bloodstream through intravenous injection [155]. PN is traditionally used during HSCT [156]. However, increasing evidence has demonstrated that EN leads to better post-transplant outcomes, such as reduced overall survival, infection and aGVHD [155, 157–160]. PN results in the loss of commensal bacteria and reduces the levels of SCFAs [26, 161], while EN promotes the recovery of gut microbial diversity and restoration of SCFA-producing bacteria [161, 162]. However, more multicenter studies are needed to explore the role of the nutritional support type in modulating intestinal microbiota and to determine the optimal type for patients undergoing allo-HSCT.

In addition, lactoferrin is an iron-binding glycoprotein that is present in bodily fluids and has pleiotropic functions, including antimicrobial, anti-inflammatory and immunoregulatory activities [163]. Inoue et al. showed that the symptoms of GI GVHD disappeared after lactoferrin therapy [164, 165]. Immunoglobulin therapy was also reported to modulate the bacterial composition and improve GVHD outcomes in a murine model [166]. These findings suggest that lactoferrin and immunoglobulins might be used as microbiota intervention strategies for the prevention and treatment of GVHD, which needs to be further investigated.

### Conclusions and future directions

Substantial evidence has shown that the intestinal microbiota is critical for the regulation of intestinal homeostasis, the immune response and the pathogenesis of aGVHD after allo-HSCT. Some strategies used to modulate the intestinal microbiota, including narrow-spectrum antibiotics (such as rifaximin, cefepime and aztreonam), prebiotics (such as RS, inulin, vitamin A), probiotics (high butyrate-producing *Clostridia*) and FMT, are recommended for the clinical prevention and treatment of aGVHD. At present, the clinical applications of these strategies are only in the initial stages and need to be further evaluated. Further multicenter studies are needed to elucidate changes in the microbiota and metabolites using metagenomic and metabonomic analysis during allo-HSCT, which will contribute to the search for new targets as biomarkers for predicting aGVHD and the identification of new intervention strategies for microbiota modulation to prevent and treat aGVHD. In addition, knowledge on the cellular and molecular mechanisms by which most intestinal microbes regulate intestinal homeostasis and immune responses in aGVHD is still lacking, and these mechanisms need to be further clarified in vivo and in vitro. The ultimate goal is to establish effective and safe strategies that modulate the microbiota to improve the outcomes of patients after allo-HSCT.

## Data Availability

Not applicable.

## Abbreviations

Allo-HSCT: Allogeneic hematopoietic stem cell transplantation
aGVHD: Acute graft-versus-host disease
FMT: Fecal microbiota transplantation
GI: Gastrointestinal
IECs: Intestinal epithelial cells
TRM: Transplant-related mortality
OS: Overall survival
VRE: Vancomycin-resistant *Enterococcus*
*AKK*: *Akkermansia muciniphila*
SCFAs: Short-chain fatty acids
RS: Resistant starch
AhR: Aryl hydrocarbon receptor
3-IS: 3-Indoxyl sulfate
ICA: Indole-3-carboxaldehyde
GVL: Graft versus leukemia
TMAO: Trimethylamine N-oxide
DMB: 3,3-Dimethyl-1-butanol
APCs: Antigen-presenting cells
ISCs: Intestinal stem cells
AMPs: Antimicrobial peptides
REG3: C-type lectin regenerating islet derived protein 3
GLP-2: Glucagon-like peptide-2
R-Spo1: R-spondin1
GPR43: G protein-coupled receptor43
PRRs: Pattern recognition receptors
TLRs: Toll-like receptors
NLRs: NOD-like receptors
LPS: Lipopolysaccharide
PSA: Polysaccharide A
MAIT: Mucosal-associated invariant T
GFO: Glutamine, fiber, and oligosaccharides
MAMPs: Microbe-associated molecular patterns
rCDI: Recurrent *Clostridium* difficile infection
auto-FMT: Autologous FMT
EN: Enteral nutrition
PN: Parenteral nutrition

## References

[1] D’ Souza A FC. Current uses and outcomes of hematopoietic cell transplantation (HCT). CIBMTR summary slides. 2019; http://www.cibmtr.org.

[2] Forman SJ NR, Antin JH, Appelbaum FR, editors. Thomas’ hematopoietic cell transplantation. 5rd ed. Chichester: Wiley. 2016.

[3] Yu J, Parasuraman S, Shah A, Weisdorf D (2019). Mortality, length of stay and costs associated with acute graft-versus-host disease during hospitalization for allogeneic hematopoietic stem cell transplantation. Curr Med Res Opin.

[4] Zeiser R, Blazar BR (2017). Acute graft-versus-host disease - biologic process, prevention, and therapy. N Engl J Med.

[5] Castilla-Llorente C, Martin PJ, McDonald GB, Storer BE, Appelbaum FR, Deeg HJ (2014). Prognostic factors and outcomes of severe gastrointestinal GVHD after allogeneic hematopoietic cell transplantation. Bone Marrow Transplant.

[6] Faith JJ, Guruge JL, Charbonneau M, Subramanian S, Seedorf H, Goodman AL (2013). The long-term stability of the human gut microbiota. Science.

[7] Lozupone CA, Stombaugh JI, Gordon JI, Jansson JK, Knight R (2012). Diversity, stability and resilience of the human gut microbiota. Nature.

[8] Hansson GC (2012). Role of mucus layers in gut infection and inflammation. Curr Opin Microbiol.

[9] Salzman NH (2010). Paneth cell defensins and the regulation of the microbiome: detente at mucosal surfaces. Gut Microbes.

[10] Brown EM, Sadarangani M, Finlay BB (2013). The role of the immune system in governing host-microbe interactions in the intestine. Nat Immunol.

[11] Renz H, Brandtzaeg P, Hornef M (2011). The impact of perinatal immune development on mucosal homeostasis and chronic inflammation. Nat Rev Immunol.

[12] Markey KA, MacDonald KP, Hill GR (2014). The biology of graft-versus-host disease: experimental systems instructing clinical practice. Blood.

[13] Jones JM, Wilson R, Bealmear PM (1971). Mortality and gross pathology of secondary disease in germfree mouse radiation chimeras. Radiat Res.

[14] van Bekkum DW, Roodenburg J, Heidt PJ, van der Waaij D (1974). Mitigation of secondary disease of allogeneic mouse radiation chimeras by modification of the intestinal microflora. J Natl Cancer Inst.

[15] Beelen DW, Haralambie E, Brandt H, Linzenmeier G, Muller KD, Quabeck K (1992). Evidence that sustained growth suppression of intestinal anaerobic bacteria reduces the risk of acute graft-versus-host disease after sibling marrow transplantation. Blood.

[16] Eckburg PB, Bik EM, Bernstein CN, Purdom E, Dethlefsen L, Sargent M (2005). Diversity of the human intestinal microbial flora. Science.

[17] Ferrara JL, Levine JE, Reddy P, Holler E (2009). Graft-versus-host disease. Lancet.

[18] Jenq RR, Ubeda C, Taur Y, Menezes CC, Khanin R, Dudakov JA (2012). Regulation of intestinal inflammation by microbiota following allogeneic bone marrow transplantation. J Exp Med.

[19] Greco R, Nitti R, Mancini N, Pasciuta R, Lorentino F, Lupo-Stanghellini MT (2021). Microbiome markers are early predictors of acute GVHD in allogeneic hematopoietic stem cell transplant recipients. Blood.

[20] Peled JU, Gomes ALC, Devlin SM, Littmann ER, Taur Y, Sung AD (2020). Microbiota as predictor of mortality in allogeneic hematopoietic-cell transplantation. N Engl J Med.

[21] Han L, Zhang H, Chen S, Zhou L, Li Y, Zhao K (2019). Intestinal microbiota can predict acute graft-versus-host disease following allogeneic hematopoietic stem cell transplantation. Biol Blood Marrow Transplant.

[22] Eriguchi Y, Takashima S, Oka H, Shimoji S, Nakamura K, Uryu H (2012). Graft-versus-host disease disrupts intestinal microbial ecology by inhibiting Paneth cell production of alpha-defensins. Blood.

[23] Taur Y, Jenq RR, Perales MA, Littmann ER, Morjaria S, Ling L (2014). The effects of intestinal tract bacterial diversity on mortality following allogeneic hematopoietic stem cell transplantation. Blood.

[24] Liu C, Frank DN, Horch M, Chau S, Ir D, Horch EA (2017). Associations between acute gastrointestinal GvHD and the baseline gut microbiota of allogeneic hematopoietic stem cell transplant recipients and donors. Bone Marrow Transplant.

[25] Gavriilaki M, Sakellari I, Anagnostopoulos A, Gavriilaki E (2020). The impact of antibiotic-mediated modification of the intestinal microbiome on outcomes of allogeneic hematopoietic cell transplantation: systematic review and meta-analysis. Biol Blood Marrow Transplant.

[26] Jenq RR, Taur Y, Devlin SM, Ponce DM, Goldberg JD, Ahr KF (2015). Intestinal blautia is associated with reduced death from graft-versus-host disease. Biol Blood Marrow Transplant.

[27] Mancini N, Greco R, Pasciuta R, Barbanti MC, Pini G, Morrow OB (2017). Enteric microbiome markers as early predictors of clinical outcome in allogeneic hematopoietic stem cell transplant: results of a prospective study in adult patients. Open Forum Infect Dis..

[28] Golob JL, Pergam SA, Srinivasan S, Fiedler TL, Liu C, Garcia K (2017). Stool microbiota at neutrophil recovery is predictive for severe acute graft vs host disease after hematopoietic cell transplantation. Clin Infect Dis.

[29] Han L, Jin H, Zhou L, Zhang X, Fan Z, Dai M (2018). Intestinal microbiota at engraftment influence acute graft-versus-host disease via the Treg/Th17 balance in Allo-HSCT recipients. Front Immunol.

[30] Parco S, Benericetti G, Vascotto F, Palmisciano G (2019). Microbiome and diversity indices during blood stem cells transplantation—new perspectives?. Cent Eur J Public Health.

[31] Galloway-Pena JR, Peterson CB, Malik F, Sahasrabhojane PV, Shah DP, Brumlow CE (2019). Fecal microbiome, metabolites, and stem cell transplant outcomes: a single-center pilot study. Open Forum Infect Dis..

[32] Payen M, Nicolis I, Robin M, Michonneau D, Delannoye J, Mayeur C (2020). Functional and phylogenetic alterations in gut microbiome are linked to graft-versus-host disease severity. Blood Adv.

[33] Ilett EE, Jorgensen M, Noguera-Julian M, Norgaard JC, Daugaard G, Helleberg M (2020). Associations of the gut microbiome and clinical factors with acute GVHD in allogeneic HSCT recipients. Blood Adv.

[34] Song A, Shen N, Gan C, Luo C, Luo C, Wang J (2021). Exploration of the relationship between intestinal flora changes and gut acute graft-versus-host disease after hematopoietic stem cell transplantation. Transl Pediatr.

[35] Ingham AC, Kielsen K, Cilieborg MS, Lund O, Holmes S, Aarestrup FM (2019). Specific gut microbiome members are associated with distinct immune markers in pediatric allogeneic hematopoietic stem cell transplantation. Microbiome.

[36] Holler E, Butzhammer P, Schmid K, Hundsrucker C, Koestler J, Peter K (2014). Metagenomic analysis of the stool microbiome in patients receiving allogeneic stem cell transplantation: loss of diversity is associated with use of systemic antibiotics and more pronounced in gastrointestinal graft-versus-host disease. Biol Blood Marrow Transplant.

[37] Heimesaat MM, Nogai A, Bereswill S, Plickert R, Fischer A, Loddenkemper C (2010). MyD88/TLR9 mediated immunopathology and gut microbiota dynamics in a novel murine model of intestinal graft-versus-host disease. Gut.

[38] Biagi E, Zama D, Nastasi C, Consolandi C, Fiori J, Rampelli S (2015). Gut microbiota trajectory in pediatric patients undergoing hematopoietic SCT. Bone Marrow Transplant.

[39] Stein-Thoeringer CK, Nichols KB, Lazrak A, Docampo MD, Slingerland AE, Slingerland JB (2019). Lactose drives *Enterococcus* expansion to promote graft-versus-host disease. Science.

[40] Ubeda C, Taur Y, Jenq RR, Equinda MJ, Son T, Samstein M (2010). Vancomycin-resistant *Enterococcus* domination of intestinal microbiota is enabled by antibiotic treatment in mice and precedes bloodstream invasion in humans. J Clin Invest.

[41] Kamboj M, Chung D, Seo SK, Pamer EG, Sepkowitz KA, Jakubowski AA (2010). The changing epidemiology of vancomycin-resistant *Enterococcus* (VRE) bacteremia in allogeneic hematopoietic stem cell transplant (HSCT) recipients. Biol Blood Marrow Transplant.

[42] Simms-Waldrip TR, Sunkersett G, Coughlin LA, Savani MR, Arana C, Kim J (2017). Antibiotic-induced depletion of anti-inflammatory clostridia is associated with the development of graft-versus-host disease in pediatric stem cell transplantation patients. Biol Blood Marrow Transplant.

[43] Mathewson ND, Jenq R, Mathew AV, Koenigsknecht M, Hanash A, Toubai T (2016). Gut microbiome-derived metabolites modulate intestinal epithelial cell damage and mitigate graft-versus-host disease. Nat Immunol.

[44] Biagi E, Zama D, Rampelli S, Turroni S, Brigidi P, Consolandi C (2019). Early gut microbiota signature of aGvHD in children given allogeneic hematopoietic cell transplantation for hematological disorders. BMC Med Genomics.

[45] Doki N, Suyama M, Sasajima S, Ota J, Igarashi A, Mimura I (2017). Clinical impact of pre-transplant gut microbial diversity on outcomes of allogeneic hematopoietic stem cell transplantation. Ann Hematol.

[46] Sofi MH, Wu Y, Ticer T, Schutt S, Bastian D, Choi HJ (2021). A single strain of *Bacteroides fragilis* protects gut integrity and reduces GVHD. JCI Insight..

[47] Shono Y, Docampo MD, Peled JU, Perobelli SM, Velardi E, Tsai JJ (2016). Increased GVHD-related mortality with broad-spectrum antibiotic use after allogeneic hematopoietic stem cell transplantation in human patients and mice. Sci Transl Med..

[48] Rooks MG, Garrett WS (2016). Gut microbiota, metabolites and host immunity. Nat Rev Immunol.

[49] Romick-Rosendale LE, Haslam DB, Lane A, Denson L, Lake K, Wilkey A (2018). Antibiotic exposure and reduced short chain fatty acid production after hematopoietic stem cell transplant. Biol Blood Marrow Transplant.

[50] Haak BW, Littmann ER, Chaubard JL, Pickard AJ, Fontana E, Adhi F (2018). Impact of gut colonization with butyrate-producing microbiota on respiratory viral infection following allo-HCT. Blood.

[51] Holtan SG, Hoeschen AL, Cao Q, Arora M, Bachanova V, Brunstein CG (2020). Facilitating resolution of life-threatening acute GVHD with human chorionic gonadotropin and epidermal growth factor. Blood Adv.

[52] Fujiwara H, Docampo MD, Riwes M, Peltier D, Toubai T, Henig I (2018). Microbial metabolite sensor GPR43 controls severity of experimental GVHD. Nat Commun.

[53] Venkataraman A, Sieber JR, Schmidt AW, Waldron C, Theis KR, Schmidt TM (2016). Variable responses of human microbiomes to dietary supplementation with resistant starch. Microbiome.

[54] Michonneau D, Latis E, Curis E, Dubouchet L, Ramamoorthy S, Ingram B (2019). Metabolomics analysis of human acute graft-versus-host disease reveals changes in host and microbiota-derived metabolites. Nat Commun.

[55] Weber D, Oefner PJ, Hiergeist A, Koestler J, Gessner A, Weber M (2015). Low urinary indoxyl sulfate levels early after transplantation reflect a disrupted microbiome and are associated with poor outcome. Blood.

[56] Weber D, Oefner PJ, Dettmer K, Hiergeist A, Koestler J, Gessner A (2016). Rifaximin preserves intestinal microbiota balance in patients undergoing allogeneic stem cell transplantation. Bone Marrow Transplant.

[57] Swimm A, Giver CR, DeFilipp Z, Rangaraju S, Sharma A, Ulezko Antonova A (2018). Indoles derived from intestinal microbiota act via type I interferon signaling to limit graft-versus-host disease. Blood.

[58] Wu K, Yuan Y, Yu H, Dai X, Wang S, Sun Z (2020). The gut microbial metabolite trimethylamine N-oxide aggravates GVHD by inducing M1 macrophage polarization in mice. Blood.

[59] Harris AC, Ferrara JL, Levine JE (2013). Advances in predicting acute GVHD. Br J Haematol.

[60] Holtan SG, Pasquini M, Weisdorf DJ (2014). Acute graft-versus-host disease: a bench-to-bedside update. Blood.

[61] Peterson LW, Artis D (2014). Intestinal epithelial cells: regulators of barrier function and immune homeostasis. Nat Rev Immunol.

[62] Pelaseyed T, Bergstrom JH, Gustafsson JK, Ermund A, Birchenough GM, Schutte A (2014). The mucus and mucins of the goblet cells and enterocytes provide the first defense line of the gastrointestinal tract and interact with the immune system. Immunol Rev.

[63] Ara T, Hashimoto D, Hayase E, Noizat C, Kikuchi R, Hasegawa Y (2020). Intestinal goblet cells protect against GVHD after allogeneic stem cell transplantation via Lypd8. Sci Transl Med..

[64] Haney EF, Mansour SC, Hancock RE (2017). Antimicrobial peptides: an introduction. Methods Mol Biol.

[65] Bevins CL, Salzman NH (2011). Paneth cells, antimicrobial peptides and maintenance of intestinal homeostasis. Nat Rev Microbiol.

[66] Levine JE, Huber E, Hammer ST, Harris AC, Greenson JK, Braun TM (2013). Low Paneth cell numbers at onset of gastrointestinal graft-versus-host disease identify patients at high risk for nonrelapse mortality. Blood.

[67] Eriguchi Y, Nakamura K, Hashimoto D, Shimoda S, Shimono N, Akashi K (2015). Decreased secretion of Paneth cell alpha-defensins in graft-versus-host disease. Transpl Infect Dis.

[68] Ferrara JL, Harris AC, Greenson JK, Braun TM, Holler E, Teshima T (2011). Regenerating islet-derived 3-alpha is a biomarker of gastrointestinal graft-versus-host disease. Blood.

[69] Zhao D, Kim YH, Jeong S, Greenson JK, Chaudhry MS, Hoepting M (2018). Survival signal REG3alpha prevents crypt apoptosis to control acute gastrointestinal graft-versus-host disease. J Clin Invest.

[70] Norona J, Apostolova P, Schmidt D, Ihlemann R, Reischmann N, Taylor G (2020). Glucagon-like peptide 2 for intestinal stem cell and Paneth cell repair during graft-versus-host disease in mice and humans. Blood.

[71] Fu YY, Egorova A, Sobieski C, Kuttiyara J, Calafiore M, Takashima S (2019). T cell recruitment to the intestinal stem cell compartment drives immune-mediated intestinal damage after allogeneic transplantation. Immunity..

[72] Takashima S, Kadowaki M, Aoyama K, Koyama M, Oshima T, Tomizuka K (2011). The Wnt agonist R-spondin1 regulates systemic graft-versus-host disease by protecting intestinal stem cells. J Exp Med.

[73] Hayase E, Hashimoto D, Nakamura K, Noizat C, Ogasawara R, Takahashi S (2017). R-Spondin1 expands Paneth cells and prevents dysbiosis induced by graft-versus-host disease. J Exp Med.

[74] Joly AL, Deepti A, Seignez A, Goloudina A, Hebrard S, Schmitt E (2016). The HSP90 inhibitor, 17AAG, protects the intestinal stem cell niche and inhibits graft versus host disease development. Oncogene.

[75] Zhou Z, Shang T, Li X, Zhu H, Qi YB, Zhao X (2020). Protecting intestinal microenvironment alleviates acute graft-versus-host disease. Front Physiol..

[76] Schluter J, Peled JU, Taylor BP, Markey KA, Smith M, Taur Y (2020). The gut microbiota is associated with immune cell dynamics in humans. Nature.

[77] Tu S, Zhong D, Xie W, Huang W, Jiang Y, Li Y (2016). Role of toll-like receptor signaling in the pathogenesis of graft-versus-host diseases. Int J Mol Sci..

[78] Cooke KR, Gerbitz A, Crawford JM, Teshima T, Hill GR, Tesolin A (2001). LPS antagonism reduces graft-versus-host disease and preserves graft-versus-leukemia activity after experimental bone marrow transplantation. J Clin Invest.

[79] Elmaagacli AH, Koldehoff M, Hindahl H, Steckel NK, Trenschel R, Peceny R (2006). Mutations in innate immune system NOD2/CARD 15 and TLR-4 (Thr399Ile) genes influence the risk for severe acute graft-versus-host disease in patients who underwent an allogeneic transplantation. Transplantation.

[80] Lorenz E, Schwartz DA, Martin PJ, Gooley T, Lin MT, Chien JW (2001). Association of TLR4 mutations and the risk for acute GVHD after HLA-matched-sibling hematopoietic stem cell transplantation. Biol Blood Marrow Transplant.

[81] Zhao Y, Liu Q, Yang L, He D, Wang L, Tian J (2013). TLR4 inactivation protects from graft-versus-host disease after allogeneic hematopoietic stem cell transplantation. Cell Mol Immunol.

[82] Imado T, Iwasaki T, Kitano S, Satake A, Kuroiwa T, Tsunemi S (2010). The protective role of host Toll-like receptor-4 in acute graft-versus-host disease. Transplantation.

[83] Hossain MS, Jaye DL, Pollack BP, Farris AB, Tselanyane ML, David E (2011). Flagellin, a TLR5 agonist, reduces graft-versus-host disease in allogeneic hematopoietic stem cell transplantation recipients while enhancing antiviral immunity. J Immunol.

[84] Jasperson LK, Bucher C, Panoskaltsis-Mortari A, Mellor AL, Munn DH, Blazar BR (2009). Inducing the tryptophan catabolic pathway, indoleamine 2,3-dioxygenase (IDO), for suppression of graft-versus-host disease (GVHD) lethality. Blood.

[85] Taylor PA, Ehrhardt MJ, Lees CJ, Panoskaltsis-Mortari A, Krieg AM, Sharpe AH (2008). TLR agonists regulate alloresponses and uncover a critical role for donor APCs in allogeneic bone marrow rejection. Blood.

[86] Penack O, Smith OM, Cunningham-Bussel A, Liu X, Rao U, Yim N (2009). NOD2 regulates hematopoietic cell function during graft-versus-host disease. J Exp Med.

[87] Schwab L, Goroncy L, Palaniyandi S, Gautam S, Triantafyllopoulou A, Mocsai A (2014). Neutrophil granulocytes recruited upon translocation of intestinal bacteria enhance graft-versus-host disease via tissue damage. Nat Med.

[88] Hulsdunker J, Ottmuller KJ, Neeff HP, Koyama M, Gao Z, Thomas OS (2018). Neutrophils provide cellular communication between ileum and mesenteric lymph nodes at graft-versus-host disease onset. Blood.

[89] Hulsdunker J, Thomas OS, Haring E, Unger S, Gonzalo Nunez N, Tugues S (2019). Immunization against poly-N-acetylglucosamine reduces neutrophil activation and GVHD while sparing microbial diversity. Proc Natl Acad Sci U S A.

[90] Hanash AM, Dudakov JA, Hua G, O'Connor MH, Young LF, Singer NV (2012). Interleukin-22 protects intestinal stem cells from immune-mediated tissue damage and regulates sensitivity to graft versus host disease. Immunity.

[91] Munneke JM, Bjorklund AT, Mjosberg JM, Garming-Legert K, Bernink JH, Blom B (2014). Activated innate lymphoid cells are associated with a reduced susceptibility to graft-versus-host disease. Blood.

[92] Lindemans CA, Calafiore M, Mertelsmann AM, O'Connor MH, Dudakov JA, Jenq RR (2015). Interleukin-22 promotes intestinal-stem-cell-mediated epithelial regeneration. Nature.

[93] Qiu J, Heller JJ, Guo X, Chen ZM, Fish K, Fu YX (2012). The aryl hydrocarbon receptor regulates gut immunity through modulation of innate lymphoid cells. Immunity.

[94] Zelante T, Iannitti RG, Cunha C, De Luca A, Giovannini G, Pieraccini G (2013). Tryptophan catabolites from microbiota engage aryl hydrocarbon receptor and balance mucosal reactivity via interleukin-22. Immunity.

[95] Behnsen J, Raffatellu M (2013). Keeping the peace: aryl hydrocarbon receptor signaling modulates the mucosal microbiota. Immunity.

[96] Wesemann DR, Portuguese AJ, Meyers RM, Gallagher MP, Cluff-Jones K, Magee JM (2013). Microbial colonization influences early B-lineage development in the gut lamina propria. Nature.

[97] Belkaid Y, Harrison OJ (2017). Homeostatic immunity and the microbiota. Immunity.

[98] Koyama M, Mukhopadhyay P, Schuster IS, Henden AS, Hulsdunker J, Varelias A (2019). MHC class II antigen presentation by the intestinal epithelium initiates graft-versus-host disease and is influenced by the microbiota. Immunity..

[99] Furusawa Y, Obata Y, Fukuda S, Endo TA, Nakato G, Takahashi D (2013). Commensal microbe-derived butyrate induces the differentiation of colonic regulatory T cells. Nature.

[100] Smith PM, Howitt MR, Panikov N, Michaud M, Gallini CA, Bohlooly YM (2013). The microbial metabolites, short-chain fatty acids, regulate colonic Treg cell homeostasis. Science.

[101] Bhattacharyya A, Hanafi LA, Sheih A, Golob JL, Srinivasan S, Boeckh MJ (2018). Graft-derived reconstitution of mucosal-associated invariant T cells after allogeneic hematopoietic cell transplantation. Biol Blood Marrow Transplant.

[102] Varelias A, Bunting MD, Ormerod KL, Koyama M, Olver SD, Straube J (2018). Recipient mucosal-associated invariant T cells control GVHD within the colon. J Clin Invest.

[103] Varelias A, Ormerod KL, Bunting MD, Koyama M, Gartlan KH, Kuns RD (2017). Acute graft-versus-host disease is regulated by an IL-17-sensitive microbiome. Blood.

[104] Jimenez-Jorge S, Labrador-Herrera G, Rosso-Fernandez CM, Rodriguez-Torres N, Pachon-Ibanez ME, Smani Y (2020). Assessing the impact on intestinal microbiome and clinical outcomes of antibiotherapy optimisation strategies in haematopoietic stem cell transplant recipients: study protocol for the prospective multicentre OptimBioma study. BMJ Open..

[105] Yoshifuji K, Inamoto K, Kiridoshi Y, Takeshita K, Sasajima S, Shiraishi Y (2020). Prebiotics protect against acute graft-versus-host disease and preserve the gut microbiota in stem cell transplantation. Blood Adv.

[106] Andermann TM, Fouladi F, Tamburini FB, Sahaf B, Tkachenko E, Greene C (2021). A fructo-oligosaccharide prebiotic is well tolerated in adults undergoing allogeneic hematopoietic stem cell transplantation: a phase I dose-escalation trial. Transplant Cell Ther..

[107] da Gama Torres HO, Vilela EG, da Cunha AS, Goulart EM, Souza MH, Aguirre AC (2008). Efficacy of glutamine-supplemented parenteral nutrition on short-term survival following allo-SCT: a randomized study. Bone Marrow Transplant.

[108] Gorshein E, Wei C, Ambrosy S, Budney S, Vivas J, Shenkerman A (2017). Lactobacillus rhamnosus GG probiotic enteric regimen does not appreciably alter the gut microbiome or provide protection against GVHD after allogeneic hematopoietic stem cell transplantation. Clin Transplant..

[109] Ladas EJ, Bhatia M, Chen L, Sandler E, Petrovic A, Berman DM (2016). The safety and feasibility of probiotics in children and adolescents undergoing hematopoietic cell transplantation. Bone Marrow Transplant.

[110] Kakihana K, Fujioka Y, Suda W, Najima Y, Kuwata G, Sasajima S (2016). Fecal microbiota transplantation for patients with steroid-resistant acute graft-versus-host disease of the gut. Blood.

[111] Spindelboeck W, Schulz E, Uhl B, Kashofer K, Aigelsreiter A, Zinke-Cerwenka W (2017). Repeated fecal microbiota transplantations attenuate diarrhea and lead to sustained changes in the fecal microbiota in acute, refractory gastrointestinal graft-versus-host-disease. Haematologica.

[112] Qi X, Li X, Zhao Y, Wu X, Chen F, Ma X (2018). Treating steroid refractory intestinal acute graft-vs.-host disease with fecal microbiota transplantation: a pilot study. Front Immunol..

[113] Kaito S, Toya T, Yoshifuji K, Kurosawa S, Inamoto K, Takeshita K (2018). Fecal microbiota transplantation with frozen capsules for a patient with refractory acute gut graft-versus-host disease. Blood Adv.

[114] Biernat MM, Urbaniak-Kujda D, Dybko J, Kapelko-Slowik K, Prajs I, Wrobel T (2020). Fecal microbiota transplantation in the treatment of intestinal steroid-resistant graft-versus-host disease: two case reports and a review of the literature. J Int Med Res.

[115] Mao D, Jiang Q, Sun Y, Mao Y, Guo L, Zhang Y (2020). Treatment of intestinal graft-versus-host disease with unrelated donor fecal microbiota transplantation capsules: a case report. Medicine (Baltimore)..

[116] van Lier YF, Davids M, Haverkate NJE, de Groot PF, Donker ML, Meijer E (2020). Donor fecal microbiota transplantation ameliorates intestinal graft-versus-host disease in allogeneic hematopoietic cell transplant recipients. Sci Transl Med..

[117] Zhao Y, Li X, Zhou Y, Gao J, Jiao Y, Zhu B (2021). Safety and efficacy of fecal microbiota transplantation for grade IV steroid refractory GI-GvHD patients: interim results from FMT2017002 trial. Front Immunol..

[118] Bilinski J, Lis K, Tomaszewska A, Grzesiowski P, Dzieciatkowski T, Tyszka M (2021). Fecal microbiota transplantation in patients with acute and chronic graft-versus-host disease-spectrum of responses and safety profile. Results from a prospective, multicenter study. Am J Hematol..

[119] Elgarten CW, Li Y, Getz KD, Hemmer M, Huang YV, Hall M (2021). Broad spectrum antibiotics and risk of graft-versus-host disease in pediatric patients transplanted for acute leukemia: association of carbapenem use with risk of acute GVHD. Transplant Cell Ther..

[120] Farowski F, Bucker V, Vehreschild JJ, Biehl L, Cruz-Aguilar R, Scheid C (2018). Impact of choice, timing, sequence and combination of broad-spectrum antibiotics on the outcome of allogeneic haematopoietic stem cell transplantation. Bone Marrow Transplant.

[121] Tanaka JS, Young RR, Heston SM, Jenkins K, Spees LP, Sung AD (2020). Anaerobic antibiotics and the risk of graft-versus-host disease after allogeneic hematopoietic stem cell transplantation. Biol Blood Marrow Transplant.

[122] Hidaka D, Hayase E, Shiratori S, Hasegawa Y, Ishio T, Tateno T (2018). The association between the incidence of intestinal graft-vs-host disease and antibiotic use after allogeneic hematopoietic stem cell transplantation. Clin Transplant..

[123] Lee SE, Lim JY, Ryu DB, Kim TW, Park SS, Jeon YW (2019). Alteration of the intestinal microbiota by broad-spectrum antibiotic use correlates with the occurrence of intestinal graft-versus-host disease. Biol Blood Marrow Transplant.

[124] Nishi K, Kanda J, Hishizawa M, Kitano T, Kondo T, Yamashita K (2018). Impact of the use and type of antibiotics on acute graft-versus-host disease. Biol Blood Marrow Transplant.

[125] Weber D, Hiergeist A, Weber M, Dettmer K, Wolff D, Hahn J (2019). Detrimental effect of broad-spectrum antibiotics on intestinal microbiome diversity in patients after allogeneic stem cell transplantation: lack of commensal sparing antibiotics. Clin Infect Dis.

[126] Routy B, Letendre C, Enot D, Chenard-Poirier M, Mehraj V, Seguin NC (2017). The influence of gut-decontamination prophylactic antibiotics on acute graft-versus-host disease and survival following allogeneic hematopoietic stem cell transplantation. Oncoimmunology..

[127] Weber D, Jenq RR, Peled JU, Taur Y, Hiergeist A, Koestler J (2017). Microbiota disruption induced by early use of broad-spectrum antibiotics is an independent risk factor of outcome after allogeneic stem cell transplantation. Biol Blood Marrow Transplant.

[128] Taur Y, Xavier JB, Lipuma L, Ubeda C, Goldberg J, Gobourne A (2012). Intestinal domination and the risk of bacteremia in patients undergoing allogeneic hematopoietic stem cell transplantation. Clin Infect Dis.

[129] Cotter PD, Ross RP, Hill C (2013). Bacteriocins - a viable alternative to antibiotics?. Nat Rev Microbiol.

[130] Lazzaro BP, Zasloff M, Rolff J (2020). Antimicrobial peptides: application informed by evolution. Science..

[131] Ting SY, Martinez-Garcia E, Huang S, Bertolli SK, Kelly KA, Cutler KJ (2020). Targeted depletion of bacteria from mixed populations by programmable adhesion with antagonistic competitor cells. Cell Host Microbe..

[132] Iyama S, Sato T, Tatsumi H, Hashimoto A, Tatekoshi A, Kamihara Y (2014). Efficacy of enteral supplementation enriched with glutamine, fiber, and oligosaccharide on mucosal injury following hematopoietic stem cell transplantation. Case Rep Oncol.

[133] Song EK, Yim JM, Yim JY, Song MY, Rho HW, Yim SK (2013). Glutamine protects mice from acute graft-versus-host disease (aGVHD). Biochem Biophys Res Commun.

[134] Noth R, Hasler R, Stuber E, Ellrichmann M, Schafer H, Geismann C (2013). Oral glutamine supplementation improves intestinal permeability dysfunction in a murine acute graft-vs-host disease model. Am J Physiol Gastrointest Liver Physiol..

[135] Crowther M, Avenell A, Culligan DJ (2009). Systematic review and meta-analyses of studies of glutamine supplementation in haematopoietic stem cell transplantation. Bone Marrow Transplant.

[136] Lounder DT, Khandelwal P, Dandoy CE, Jodele S, Grimley MS, Wallace G (2017). Lower levels of vitamin A are associated with increased gastrointestinal graft-versus-host disease in children. Blood.

[137] Sharma A, Tilak T, Bakhshi S, Raina V, Kumar L, Chaudhary S (2016). Lactobacillus brevis CD2 lozenges prevent oral mucositis in patients undergoing high dose chemotherapy followed by haematopoietic stem cell transplantation. ESMO Open..

[138] Gerbitz A, Schultz M, Wilke A, Linde HJ, Scholmerich J, Andreesen R (2004). Probiotic effects on experimental graft-versus-host disease: let them eat yogurt. Blood.

[139] Zmora N, Zilberman-Schapira G, Suez J, Mor U, Dori-Bachash M, Bashiardes S (2018). Personalized gut mucosal colonization resistance to empiric probiotics is associated with unique host and microbiome features. Cell..

[140] Cohen SA, Woodfield MC, Boyle N, Stednick Z, Boeckh M, Pergam SA (2016). Incidence and outcomes of bloodstream infections among hematopoietic cell transplant recipients from species commonly reported to be in over-the-counter probiotic formulations. Transpl Infect Dis.

[141] Tsilingiri K, Rescigno M (2013). Postbiotics: what else?. Benef Microbes.

[142] Mayorgas A, Dotti I, Salas A (2021). Microbial metabolites, postbiotics, and intestinal epithelial function. Mol Nutr Food Res..

[143] Riwes M, Reddy P (2020). Short chain fatty acids: postbiotics/metabolites and graft versus host disease colitis. Semin Hematol.

[144] Ott SJ, Waetzig GH, Rehman A, Moltzau-Anderson J, Bharti R, Grasis JA (2017). Efficacy of sterile fecal filtrate transfer for treating patients with clostridium difficile infection. Gastroenterology..

[145] Khoruts A, Sadowsky MJ (2016). Understanding the mechanisms of faecal microbiota transplantation. Nat Rev Gastroenterol Hepatol.

[146] Surawicz CM, Brandt LJ, Binion DG, Ananthakrishnan AN, Curry SR, Gilligan PH (2013). Guidelines for diagnosis, treatment, and prevention of Clostridium difficile infections. Am J Gastroenterol..

[147] Goeser F, Sifft B, Stein-Thoeringer C, Farowski F, Strassburg CP, Brossart P (2021). Fecal microbiota transfer for refractory intestinal graft-versus-host disease—experience from two German tertiary centers. Eur J Haematol.

[148] Dailey FE, Turse EP, Daglilar E, Tahan V (2019). The dirty aspects of fecal microbiota transplantation: a review of its adverse effects and complications. Curr Opin Pharmacol.

[149] DeFilipp Z, Bloom PP, Torres Soto M, Mansour MK, Sater MRA, Huntley MH (2019). Drug-resistant *E. coli* bacteremia transmitted by fecal microbiota transplant. N Engl J Med..

[150] Cammarota G, Ianiro G, Tilg H, Rajilic-Stojanovic M, Kump P, Satokari R (2017). European consensus conference on faecal microbiota transplantation in clinical practice. Gut.

[151] Taur Y, Coyte K, Schluter J, Robilotti E, Figueroa C, Gjonbalaj M (2018). Reconstitution of the gut microbiota of antibiotic-treated patients by autologous fecal microbiota transplant. Sci Transl Med..

[152] DeFilipp Z, Peled JU, Li S, Mahabamunuge J, Dagher Z, Slingerland AE (2018). Third-party fecal microbiota transplantation following allo-HCT reconstitutes microbiome diversity. Blood Adv.

[153] Ramai D, Zakhia K, Ofosu A, Ofori E, Reddy M (2019). Fecal microbiota transplantation: donor relation, fresh or frozen, delivery methods, cost-effectiveness. Ann Gastroenterol.

[154] Zhang F, Zuo T, Yeoh YK, Cheng FWT, Liu Q, Tang W (2021). Longitudinal dynamics of gut bacteriome, mycobiome and virome after fecal microbiota transplantation in graft-versus-host disease. Nat Commun.

[155] Reese MK, Hewlings S (2019). Enteral versus parenteral nutrition: use in adult patients undergoing hematopoietic stem cell transplantation. Clin J Oncol Nurs.

[156] McMillen KK, Coghlin-Dickson T, Adintori PA (2021). Optimization of nutrition support practices early after hematopoietic cell transplantation. Bone Marrow Transplant.

[157] Gonzales F, Bruno B, Alarcon Fuentes M, De Berranger E, Guimber D, Behal H (2018). Better early outcome with enteral rather than parenteral nutrition in children undergoing MAC allo-SCT. Clin Nutr..

[158] Guieze R, Lemal R, Cabrespine A, Hermet E, Tournilhac O, Combal C (2014). Enteral versus parenteral nutritional support in allogeneic haematopoietic stem-cell transplantation. Clin Nutr.

[159] Seguy D, Duhamel A, Rejeb MB, Gomez E, Buhl ND, Bruno B (2012). Better outcome of patients undergoing enteral tube feeding after myeloablative conditioning for allogeneic stem cell transplantation. Transplantation.

[160] Woods T, Tariman JD, Lee YM (2019). Enteral and parenteral nutrition: an integrative literature review on nutrition in pediatric recipients of hematopoietic stem cell transplantation. Clin J Oncol Nurs.

[161] DÁmico F, Biagi E, Rampelli S, Fiori J, Zama D, Soverini M (2019). Enteral nutrition in pediatric patients undergoing hematopoietic SCT promotes the recovery of gut microbiome homeostasis. Nutrients..

[162] Andersen S, Staudacher H, Weber N, Kennedy G, Varelias A, Banks M (2020). Pilot study investigating the effect of enteral and parenteral nutrition on the gastrointestinal microbiome post-allogeneic transplantation. Br J Haematol.

[163] Ward PP, Paz E, Conneely OM (2005). Multifunctional roles of lactoferrin: a critical overview. Cell Mol Life Sci.

[164] Inoue M, Okamura T, Yasui M, Sakata N, Yagi K, Kawa K (2001). Lactoferrin for gut GVHD. Bone Marrow Transplant.

[165] van der Velden WJ, Blijlevens NM, Donnelly JP (2008). The potential role of lactoferrin and derivatives in the management of infectious and inflammatory complications of hematology patients receiving a hematopoietic stem cell transplantation. Transpl Infect Dis.

[166] Bouazzaoui A, Huber E, Dan A, Al-Allaf FA, Pfirstinger J, Sprotte G (2017). Reduction of aGVHD using chicken antibodies directed against intestinal pathogens in a murine model. Blood.

